# Explainable deep learning and biomechanical modeling for TMJ disorder morphological risk factors

**DOI:** 10.1172/jci.insight.178578

**Published:** 2024-07-11

**Authors:** Shuchun Sun, Pei Xu, Nathan Buchweitz, Cherice N. Hill, Farhad Ahmadi, Marshall B. Wilson, Angela Mei, Xin She, Benedikt Sagl, Elizabeth H. Slate, Janice S. Lee, Yongren Wu, Hai Yao

**Affiliations:** 1 Clemson-MUSC Joint Bioengineering Program, Department of Bioengineering and; 2 School of Computing, Clemson University, Clemson, South Carolina, USA.; 3 Department of Oral Health Sciences, Medical University of South Carolina, Charleston, South Carolina, USA.; 4 Center for Clinical Research, University Clinic of Dentistry, Medical University of Vienna, Vienna, Austria.; 5 Department of Statistics, Florida State University, Tallahassee, Florida, USA.; 6 National Institute of Dental and Craniofacial Research (NIDCR), NIH, Craniofacial Anomalies and Regeneration Section, Bethesda, Maryland, USA.; 7 Department of Orthopaedics, Medical University of South Carolina, Charleston, South Carolina, USA.

**Keywords:** Bone biology, Metabolism, Cartilage, Orthopedics, Osteoarthritis

## Abstract

Clarifying multifactorial musculoskeletal disorder etiologies supports risk analysis, development of targeted prevention, and treatment modalities. Deep learning enables comprehensive risk factor identification through systematic analyses of disease data sets but does not provide sufficient context for mechanistic understanding, limiting clinical applicability for etiological investigations. Conversely, multiscale biomechanical modeling can evaluate mechanistic etiology within the relevant biomechanical and physiological context. We propose a hybrid approach combining 3D explainable deep learning and multiscale biomechanical modeling; we applied this approach to investigate temporomandibular joint (TMJ) disorder etiology by systematically identifying risk factors and elucidating mechanistic relationships between risk factors and TMJ biomechanics and mechanobiology. Our 3D convolutional neural network recognized TMJ disorder patients through participant-specific morphological features in condylar, ramus, and chin. Driven by deep learning model outputs, biomechanical modeling revealed that small mandibular size and flat condylar shape were associated with increased TMJ disorder risk through increased joint force, decreased tissue nutrient availability and cell ATP production, and increased TMJ disc strain energy density. Combining explainable deep learning and multiscale biomechanical modeling addresses the “mechanism unknown” limitation undermining translational confidence in clinical applications of deep learning and increases methodological accessibility for smaller clinical data sets by providing the crucial biomechanical context.

## Abstract 2

We combined explainable deep learning and multiscale biomechanical modeling to identify and explain subject-specific morphological risk factors in temporomandibular joint disorders.

## Graphical abstract

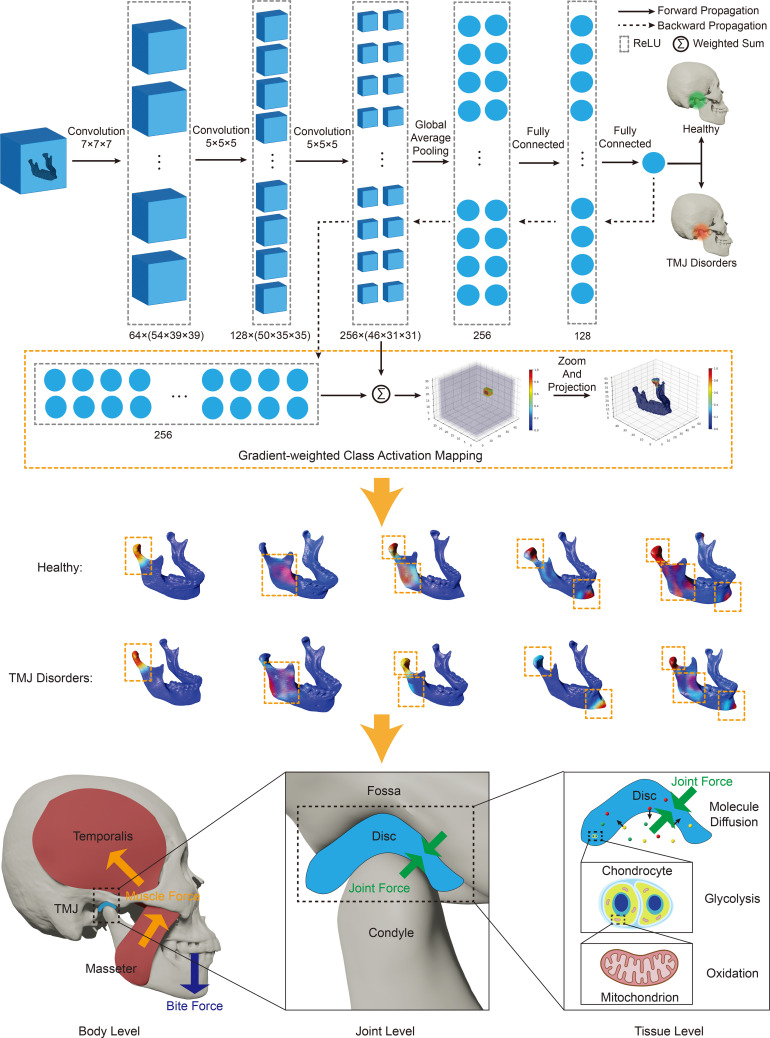

## Introduction

In the evolving landscape of biomedical research, understanding multifactorial musculoskeletal conditions has become paramount. Traditionally, many approaches are driven by single-factor, observation-based hypotheses, but human inference is subjective, highly variable, and has a limited capacity to define complex relationships. Further, integrating results from multiple studies, each investigating a discrete set of risk factors, to cohesively characterize mechanistic relationships is challenging. Understanding how and why complex disorders develop, along with how to best treat disorders, requires a more systematic and objective approach to characterize disorder patterns and associated risk.

As we transition into the modern era, disease data sets have become more accessible, offering the possibility of using data-driven approaches to enhance etiological understanding; as one such approach, machine learning has been widely used to analyze disease data sets and enhance disease diagnosis, prevention, and treatment. Machine learning algorithms enable systematic and complex multifactorial consideration, which is difficult to achieve with only subjective and qualitative observation of complex data. Despite the value of deep learning for systematic characterization of data inputs and risk factors, deep learning alone does not provide sufficient context to facilitate mechanistic understanding of model outputs or describe the biomechanical and physiological environment or root etiology. Conversely, multiscale biomechanical models can evaluate mechanistic etiology of identified risk factors and describe the joint mechanical environment, including joint mechanics, nutrient availability, and energy metabolism (1–7).

By incorporating multiscale biomechanical models in conjunction with deep learning algorithms, the deterministic relationship among morphology, mechanics, and mechanobiology can be defined within an appropriate clinical and biomechanical context; taken together, these relationships offer a powerful tool to advance musculoskeletal disorder etiological understanding, preventive care, and development of targeted treatment modalities. This paper proposes a hybrid approach combining deep learning and multiscale biomechanical modeling for investigation of musculoskeletal disorder etiologies; we explore temporomandibular joint (TMJ) disorders as an example to demonstrate the approach’s utility.

TMJ disorders affect an estimated 11.2–12.4 million adults in the United States (8) and cause substantial economic burden; individual costs average more than $1,500 per person per 6-month period (8), totaling $35–39 billion annually. Despite this high incidence and economic burden, TMJ disorder etiology is poorly understood. Clinical care for TMJ disorders is often limited to nonspecific treatment with few preventive options. Identification of major TMJ disorder risk factors and an understanding of their mechanistic relationships with TMJ biomechanical function are essential to improve patient care by informing prevention and development of targeted treatment modalities.

TMJ disorder risk is related to craniofacial morphology, emphasizing the importance of TMJ morphological evaluation in addressing the clinical need for targeted treatment. Prior studies (9–12) suggest that the combined influence of several morphological risk factors likely causes adverse TMJ mechanical environments, leading to TMJ disorders. Current characterization of the relationship between TMJ morphology, mechanics, and joint function largely relies on clinical observation of prevalent morphological patterns in patients with TMJ disorder to inform hypothesis-driven investigation (9, 10, 13). In TMJ-related research, machine learning has been extensively used for CT and MRI segmentation (14–16); its applications in characterizing TMJ morphology and assessing dysfunction risk have been limited. Patients with TMJ condylar degeneration, osteoarthritis, and disc displacement were identified with prior deep learning models, improving diagnostic screening (17–20). However, important limitations of prior models must be addressed to facilitate clinical implementation of deep learning for TMJ disorder diagnosis, risk analysis, and treatment optimization.

Prior TMJ deep learning models implemented 2D joint analyses (21), relying on 2D scaled projections of 3D structures and preventing complete representation of 3D TMJ morphology; a 3D deep learning model is necessary to improve accuracy of deep learning results. As with many traditional machine learning algorithms, these models also only provide a “black box” relationship between structure and function. This approach prevents identification of underlying structural factors driving model outputs, informed risk analysis, and etiological investigation. Instead, explainable deep learning (22) algorithms provide insight into model prediction, enabling identification of key TMJ morphological region(s) for determining TMJ disorder risk.

**Figure 1:**
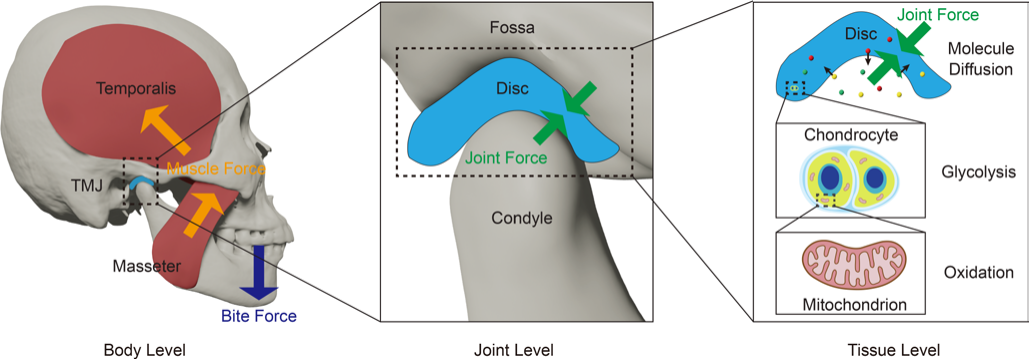
Multiscale representation of the TMJ. At the body level (left-most image), the TMJ joint force is balanced by masticatory muscle force and bite force at the teeth. At the joint level (center image), the TMJ disc absorbs the force exerted between the TMJ fossa and condyle, playing a pivotal role in joint health. At the tissue level (right-most image), the avascular nature of the TMJ disc means that it primarily relies on diffusion for nutrient and oxygen supply, a process markedly affected by joint loading. Consequently, nutrient availability and ATP production through energy metabolism are crucial for maintaining TMJ disc health. Across these scales, mandibular morphology drives TMJ joint mechanics, and joint mechanics drives the underlying biological process, emphasizing the interconnectedness of structure and function in the TMJ.

As previously described, deep learning enables systematic characterization of morphology but cannot facilitate mechanistic understanding of disorder etiology. Mechanical loading in the TMJ is determined by masticatory muscle and bite forces, which are primarily absorbed by the TMJ disc and articular cartilage. The TMJ disc plays a critical role with its mechanical response being integral to overall joint health. This disc is an avascular structure, and its nutrients are supplied solely by diffusion, a process markedly impacted by joint loading. When subjected to excessive loading, the nutrient environment within the disc can be compromised, leading to degenerative changes and TMJ disorders. Understanding this complex interplay between morphological risk factors, mechanical loading, and nutrient supply is fundamental to accurately modelling and characterizing TMJ disorders (Figure 1).

The objective of this study is to develop a systematic and reliable approach to investigate the multifactorial nature of musculoskeletal disorders by integrating explainable deep learning and multiscale biomechanical modeling; characterization of TMJ disorder morphological risk factors is used as an example demonstrating approach utility. The proposed hybrid approach enables characterization of TMJ disorder morphological risk factors within the relevant biomechanical and physiological joint environment. The developed 3D convolutional neural network derived from 3D cone beam computed tomography (CBCT) images differentiated TMJ disorder patients from healthy controls; explainable deep learning identified the mandibular condyle, ramus, and chin regions as being crucial for TMJ disorder status prediction. Morphological risk factors varied substantially between patients, emphasizing the need for individualized analysis and treatment. Based on the regions of interest identified by the deep learning model, we further analyzed effects of mandibular size and condylar shape on the TMJ biomechanical and physiological environment. Our biomechanical models showed that small mandibular size can increase joint forces, deplete nutrient availability, and cause insufficient ATP production in the TMJ disc. The models also demonstrate that condyles with a flat shape can reduce joint contact area and increase TMJ disc energy density. The high-risk patterns well explained previous incidence data and clinical observations that women and individuals with Class II skeletal malocclusion — characterized by the mandible being positioned behind the maxilla — have higher TMJ disorder risk due to their smaller mandibular sizes (23–25). Combining deep learning and multiscale biomechanical modeling resolved the well-recognized deep learning “mechanism unknown” limitation undermining translational confidence in utilizing deep learning to enhance targeted and preventive TMJ disorder care. This hybrid approach also increases methodological accessibility for smaller clinical data sets by providing the crucial biomechanical context for interpretation of deep learning results. Although demonstrated here within the context of TMJ disorders, this approach has the potential for utilization in other complex musculoskeletal systems including the knee and spine.

## Results

### Development and performance of 3D explainable convolutional neural network.

**Figure 2:**
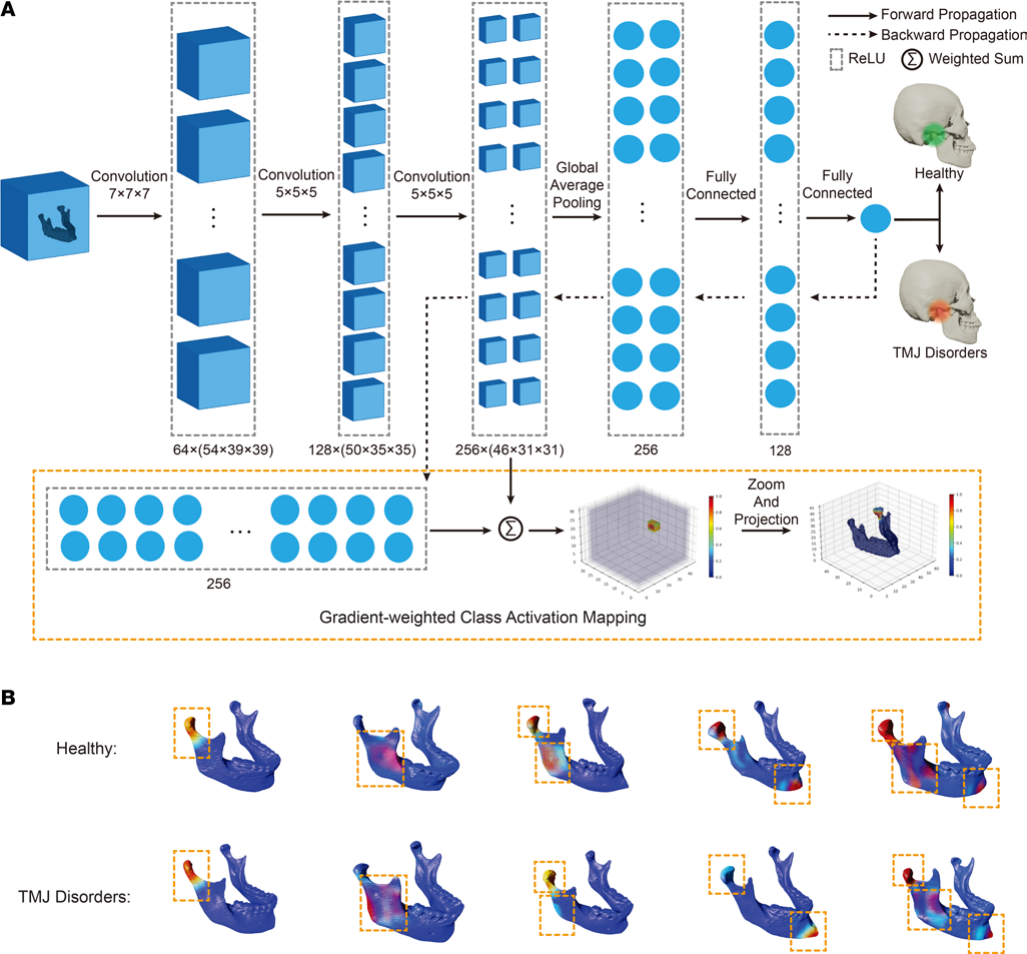
3D convolutional neural network and examples of saliency maps. (**A**) Overview of the 3D convolutional neural network approach for distinguishing between healthy participants and patients with TMJ disorder. CBCT from 80 training participants (40 healthy and 40 with TMJ disorder) and 24 validation participants (12 healthy and 12 with TMJ disorder) were utilized to train and validate this model. The CBCT images were manually segmented to obtain 3D mandible geometries, which were then voxelized into a 3D 0–1 matrix. The classification model consists of 3 3D convolutional layers, 2 fully connected layers, and a global average pooling layer. Saliency maps, generated using Grad-CAM, pinpoint the most influential regions for classification. (**B**) Examples of saliency maps were generated using Grad-CAM. 3 key regions were identified: the TMJ condyle, mandibular ramus, and chin. Each individual exhibited a unique combination of these regions, emphasizing the multifaceted nature of TMJ disorder morphological risk factors. Detailed regions of interest for each participant can be found in Supplemental Table 1.

We developed a 3D convolutional neural network, a type of deep learning model adept at analyzing complex voxel data (Figure 2A). This model was trained using data extracted from CBCT images, which provide detailed geometries of the mandibles from our sample groups, which included 40 patients with TMJ disorders and 40 people in a healthy control group (referred to as healthy controls). CBCT images from another 12 patients with TMJ disorder and 12 healthy controls were used as a validation set. The model achieved 100% and 83% accuracy in the training and validation sets, respectively. A receiver operator characteristics (ROC) analysis (26) further evaluated the model’s capacity to predict TMJ disorder status based on the sigmoid score. The model had excellent predictive capacity with an AUC of ROC of 0.952 along with high sensitivity (0.942) and specificity (0.981).

Participant-specific saliency maps were generated using the gradient-weighted class activation mapping (Grad-CAM) method (27), identifying the regions driving classification results. Saliency maps generated by Grad-CAM were compared with those generated by Grad-CAM++ to ensure consistency across different saliency map generation algorithms (Supplemental Figure 1). Three areas were identified with each individual having a participant-specific combination of regions contributing to model prediction: the TMJ condyle, mandibular ramus, and the chin (Supplemental Table 1; supplemental material available online with this article; https://doi.org/10.1172/jci.insight.178578DS1). The fact that each participant had a specific combination of 1, 2, or 3 of these areas (Figure 2B) contributing to model prediction emphasized the complex and multifactorial nature of TMJ disorder morphological risk factors.

### TMJ disorder morphological pattern identified by deep learning: mandibular size.

While our saliency maps provide critical information regarding TMJ disorder morphological patterns, direct interpretation of the underlying morphological features can be difficult. Furthermore, their utility in clinical and research settings can be enhanced by translating these features into commonly used morphometric measurements. Such a translation not only facilitates statistical analyses due to its quantifiable nature but also bolsters interpretability by directly correlating findings with clinically relevant anatomical features. Therefore, we sought to extract and quantify morphometric features identified in saliency maps for use in TMJ disorder clinical and research scenarios.

**Figure 3:**
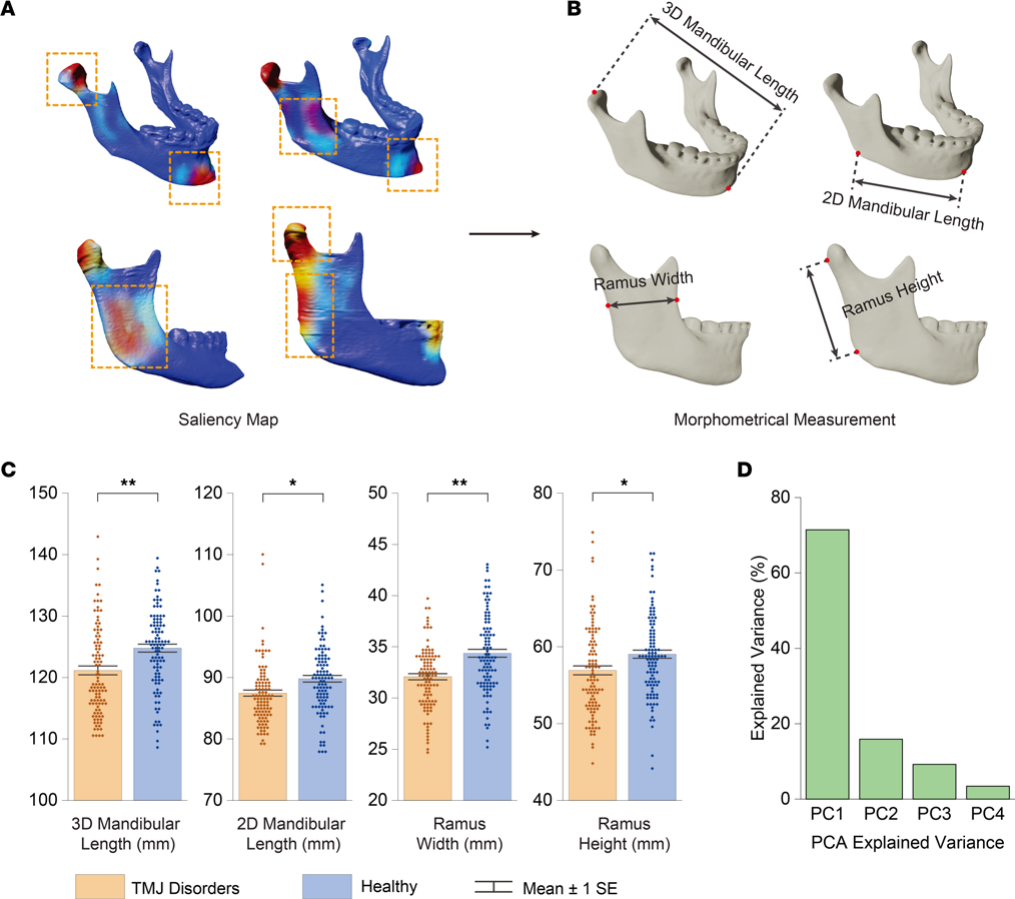
Analysis of mandibular dimensions and their association with TMJ disorders. (**A**) Saliency maps generated from deep learning models show key landmarks on the mandible, representing diverse mandibular dimensions. (**B**) Morphometric measurements extracted from saliency maps (3D mandibular length, 2D mandibular length, ramus width, and ramus height). (**C**) Comparative analysis of measurements from patients with TMJ disorder (*n* = 104 derived from 52 individuals, each contributing left and right measurements) and healthy controls (*n* = 104 derived from 52 individuals, each contributing left and right measurements) with a mixed effects model to account for the correlation between left and right side measurements within participants. The results reveal the differences between the 2 groups. In the figure, asterisks denote the level of statistical significance; * *P* < 0.05, **P < 0.01, and ***P < 0.001. (**D**) PCA results with the first principal component (PC1) accounting for 71.5% of the total variance emphasizing the ‘size’ factor in craniofacial morphology. A biplot of the PCA results can be found in Supplemental Figure 2. These analyses underscore the importance of mandibular dimensions in understanding TMJ disorders.

The saliency map reveals 3 key anatomical regions on the mandible: the TMJ condyle, ramus, and chin. Positioned at the most distant points on the mandibular structure, these regions epitomize ‘extreme’ locations. Notably, the distance between these landmarks characterizes dimensions across various parts of the mandible (Figure 3A). Consequently, we initiated our investigation by treating these landmarks as vital indicators of diverse mandibular dimensions. From the saliency maps, we extracted measurements for 3D mandibular length (depicting overall length), 2D mandibular length (denoting the distance from lateral-posterior to medial-anterior points), ramus width (representing the width of the ramus region), and ramus height (indicating the height of the ramus region) (Figure 3B). Using a mixed effects model to account for the correlation between left and right side measurements within participants, we found that patients with TMJ disorder had significantly smaller 3D mandibular length (*P* = 0.007), 2D mandibular length (*P* = 0.022), ramus width (*P* = 0.001), and ramus height (*P* = 0.047) than healthy controls (Figure 3C and Supplemental Table 2).

To fully understand the multidimensional morphological differences in mandibular dimensions, we turned to Principal Component Analysis (PCA). Given the multidimensional nature of our data, analyzing each parameter separately could overlook crucial interactions and correlations among the variables. PCA, however, condenses the information contained in several original variables into a smaller set of new composite dimensions, with each dimension orthogonal to the others. These new dimensions are derived such that they capture the maximum possible variance in the data, making them an effective tool for distilling the essential structure of our data. We performed PCA on 4 key parameters: 3D mandibular length, 2D mandibular length, ramus width, and ramus height, all of which were derived from participant-specific saliency maps. This allowed us to reduce the dimensionality of our data and gain a deeper understanding of the morphological variations contributing to TMJ disorders.

Upon normalization of the parameters, PCA elucidated that the first principal component (PC1) accounted for a substantial 71.45% of the total variance (Figure 3D), with each of the measurements showing strong correlation with PC1 (3D mandibular length: 0.9150; 2D mandibular length: 0.8799; ramus width: 0.7477; ramus height: 0.8293). This suggests that each variable substantially contributes to PC1, indicating that it predominantly represents a ‘size’ factor in craniofacial morphology. All 4 measurements also demonstrated high loadings (3D mandibular length: 0.5413; 2D mandibular length: 0.5202; ramus width: 0.4426; ramus height: 0.4905), further reinforcing the importance of PC1 (Supplemental Figure 2). Among the 4 morphological indicators, 3D mandibular length showed both the highest loading and strongest correlation with PC1, proving its efficacy to represent mandibular size.

### TMJ disorder morphological pattern identified by deep learning: TMJ condyle size and shape.

**Figure 4:**
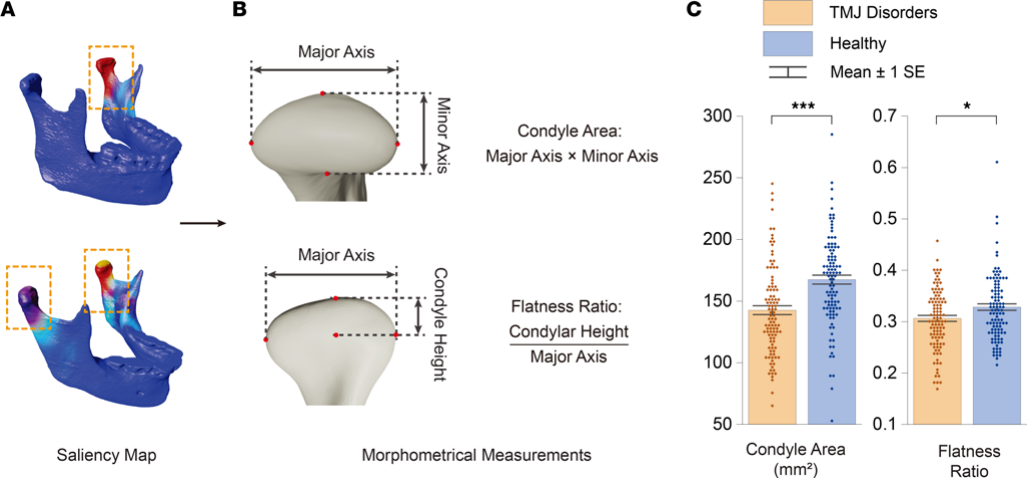
TMJ condyle morphology and its correlation with TMJ disorders. (**A**) Saliency maps generated from deep learning models highlight the TMJ condyle as an area of interest, with variations observed in the labeling of one or both condyles among individuals. (**B**) Detailed assessment of the condyle’s size and shape within the study cohort, differentiating between the overall dimensions (large or small) and the flatness (steep or flat). (**C**) Comparative analysis of measurements from patients with TMJ disorder (*n* = 104 derived from 52 participants, each contributing left and right measurements) and healthy control measurements (*n* = 104 derived from 52 participants, each contributing left and right measurements) with a mixed effects model to account for the correlation between left and right side measurements within individuals. The results reveal that patients with TMJ disorder tend to have smaller condyle areas and a more flattened condyle shape in contrast with healthy controls. In the figure, asterisks denote the level of statistical significance, *P < 0.05, **P < 0.01, and ***P < 0.001. These insights underscore the role of condyle morphology in the context of TMJ disorders.

In addition to evaluating mandibular size through morphometric indicators, the saliency map directed our attention to intrajoint morphological features, specifically regarding the TMJ condyle. The condyle is of great importance in the context of TMJ disorders due to its critical role in the mechanical function and stability of the joint (Figure 4A). Changes in the size and shape of the condyle can alter the load distribution and biomechanical environment within the TMJ, potentially leading to pathological conditions such as TMJ disorders. Consequently, we examined condyle size and shape in our cohort (Figure 4B). Using a mixed effects model to account for the correlation between left and right side measurements within participants, we found that patients with TMJ disorder exhibited smaller condyle area (*P* < 0.001) and flatter condyle shape (*P* = 0.046) than healthy controls (Figure 4C), suggesting a morphological link to TMJ disorder risk.

For both the mandibular size and condyle size and shape, the results highlight the effectiveness of our deep learning model as it accurately identifies key morphological patterns related to TMJ disorders, proving the power and precision of our explainable deep learning algorithm. We found that patients with TMJ disorders have smaller mandible size, smaller condyle area, and flatter condyle shape compared with healthy controls. These findings provide insight into morphological characteristics of TMJ disorders and provide a foundation for further mechanistic analysis using computational modeling.

### Effect of mandibular size on forces exerted on the TMJ.

**Figure 5:**
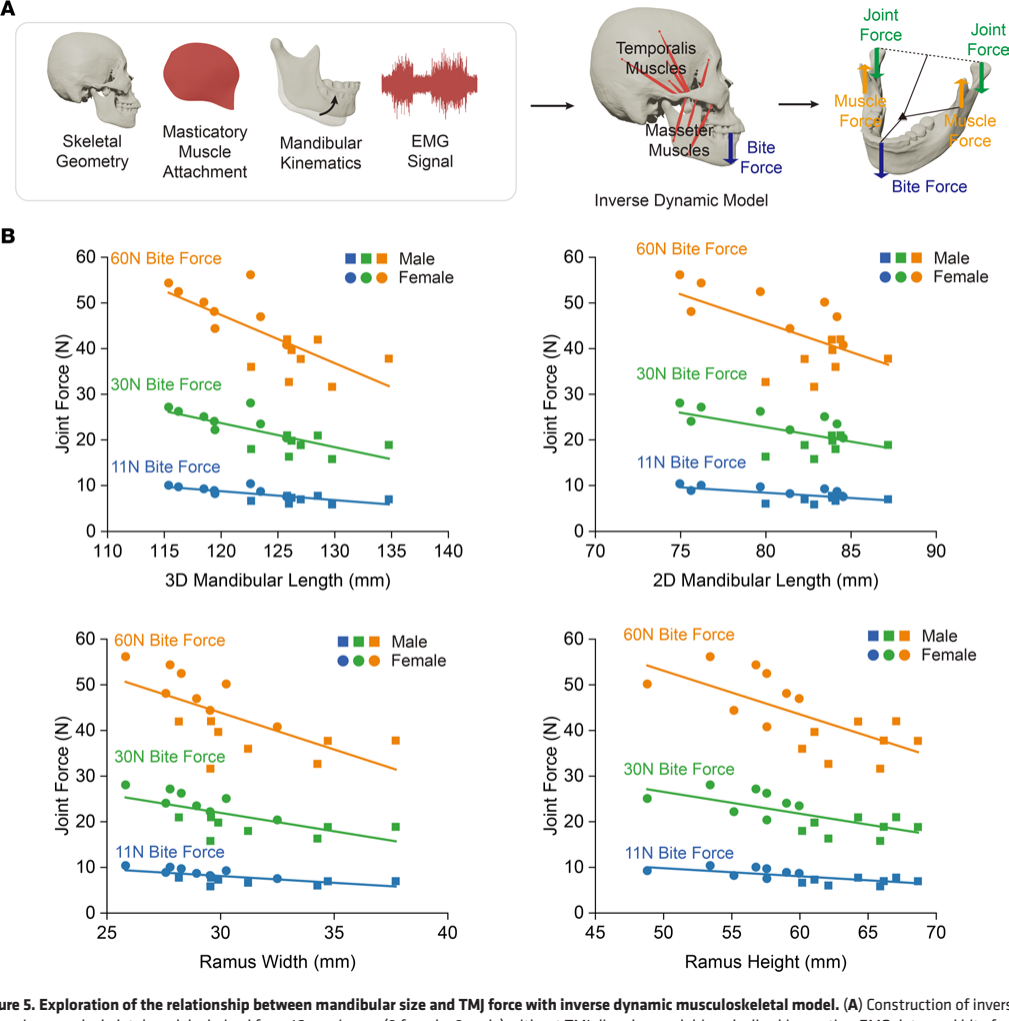
Exploration of the relationship between mandibular size and TMJ force with inverse dynamic musculoskeletal model. (**A**) Construction of inverse dynamic musculoskeletal models derived from 16 specimens (8 female, 8 male) without TMJ disorders and driven by live kinematics, EMG data, and bite forces from reference participants (Supplemental Figure 4). 3 bite force levels (11 N, 30 N, and 60 N) simulate varying bite force scenarios, with magnitudes based on human bite forces during consumption of foods with diverse textures. (**B**) Analysis of morphological indicators, such as 3D and 2D mandibular lengths, ramus width, and height, shows a negative correlation with TMJ joint force across bite force levels of 11N, 30N, and 60N (*n* = 16, 8 males and 8 females). Specifically, participants with larger mandibles tend to have reduced joint forces at a given bite force level. This trend is statistically significant, as seen in the correlations for 3D mandibular length (11 N, 30 N, and 60 N: *P* = 0.001, R²=0.5340), 2D mandibular length (11 N, 30 N, 60 N: *P* = 0.014, R²=0.3627), ramus width (11 N, 30 N, 60 N: *P* = 0.005, R²=0.4382), and ramus height (11 N, 30 N, 60 N: *P* = 0.003, R²=0.4719). Solid lines represent curve fittings where the differences are statistically significant (*P* < 0.05). Further, the moment arm ratio, which dictates the relationship between muscle force and bite force, suggests nonproportional scaling in the human mandibular musculoskeletal system (see Supplemental Figure 5). Specifically, individuals with smaller mandibles have smaller muscle force and bite force moment arm ratios, leading to increased joint forces at a given bite force. This observation aligns with clinical findings that link smaller mandibular sizes in females and patients with class II dentofacial deformity to a higher risk of TMJ disorders due to elevated joint forces.

Since the human mandible operates as an integrated unit, variations in structural features, such as size, can influence the force exerted on the TMJ. To explore this relationship, we constructed participant-specific models using inverse dynamic musculoskeletal modeling. These models were developed using a separate data set of 16 scans (8 female and 8 male) of the heads of cadavers who, in life, did not have TMJ disorder, enabling consideration of parameters that cannot be ethically obtained in vivo and reducing functional variability to clarify mechanistic relationships. These cadaveric models were driven by mandible kinematics, EMG data, and bite forces sourced from 2 specific live reference participants. Male cadaveric models were driven by data from the male reference participant, while female cadaveric models were driven by data from the female reference participant. Utilizing data from only 2 reference participants allowed us to minimize external influences and focus exclusively on morphological factors. For each model, we used 3 bite force levels (11N, 30N, and 60N) to simulate small, medium, and large bite forces within our multibody model (28). The range of bite force magnitudes was determined by referring to human bite forces used in chewing typical solid foods with various textures (29) (Figure 5A).

All size-related morphological indicators (3D mandibular length, 2D mandibular length, ramus width, and ramus height) were negatively correlated with TMJ reaction force at all bite force levels. The relationship between bite force and joint force is determined by the moment arm ratio between muscle force and bite force. By comparing the morphological indicators related to mandibular size with the moment arm ratio, we found that the human mandibular musculoskeletal system does not scale proportionally; people with small mandibles tend to have small moment arm ratios, increasing joint force at the same bite force level (Supplemental Figure 5). Our result well explained with the clinical observation that women and patients with Class II dentofacial deformities, who typically have smaller mandibular size and tend to have larger joint force, are more likely to have TMJ disorders (23–25) (Figure 5B).

### Effect of mandibular size on biological responses in the TMJ disc.

**Figure 6:**
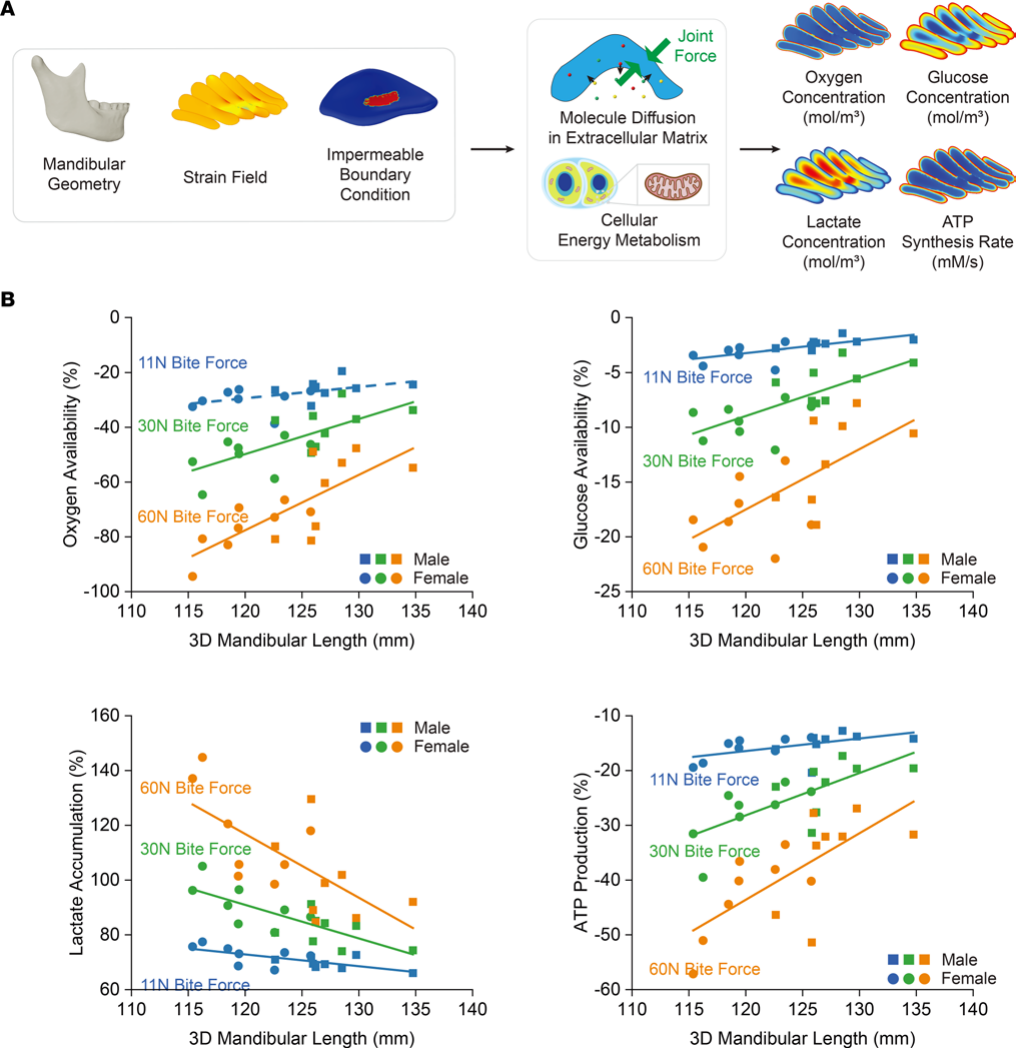
Exploration of the TMJ disc’s biological responses to mechanical loading and its relationship with mandibular size with solute diffusion and energy metabolism model. (**A**) Implementation of solute diffusion and energy metabolism models to assess the impact of mandibular size on TMJ disc oxygen and glucose availability, lactate accumulation, and ATP production. Simulation of TMJ disc compression during static biting reveals the nutrient environment’s response to localized solute exchange reduction in the contact area and mechanical strain–dependent solute diffusion in the disc’s loading volume. Solute movements are dictated by Fick’s second law, with oxygen, glucose consumption rates, and ATP production determined by the stoichiometry of intracellular energy metabolic reactions. (**B**) Analysis indicates that participants with smaller mandibles and larger joint forces experience compromised TMJ disc nutrient availability, increased lactate accumulation, and reduced ATP production (*n* = 16, 8 males and 8 females). 3D mandibular length exhibits the strongest correlation with these mechanobiological indicators. Oxygen versus mandibular length (11 N: *P* = 0.052, R² = 0.2441; 30 N: *P* = 0.002, R² = 0.4991; 60 N: *P* = 0.001, R² = 0.5906). Glucose versus mandibular length (11 N: *P* = 0.004, R² = 0.4507; 30 N: *P* = 0.002, R² = 0.5246; 60 N: *P* = 0.006, R² = 0.4347). Lactate versus mandibular length (11 N: *P* = 0.004, R² = 0.4628; 30 N: *P* = 0.001, R²=0.5753; 60 N: *P* = 0.004, R² = 0.4520). ATP versus mandibular length (11 N: *P* = 0.029, R² = 0.2960; 30 N: *P* = 0.002, R² = 0.5042; 60 N: *P* = 0.002, R² = 0.4951). Solid lines represent curve fittings where the differences are statistically significant (*P* < 0.05), and dashed lines represent curve fittings where the differences are not statistically significant (*P* ≥ 0.05). Elevated joint forces can lead to an expanded TMJ disc–condyle contact area and heightened mechanical strain, impeding nutrient transport and metabolic waste removal within the TMJ disc (Supplemental Figure 7). This model offers a mechanistic explanation for the increased susceptibility to degenerative changes in individuals with smaller mandibles.

In addition to the mechanical analysis, we also built models to explore the TMJ disc’s biological response to mechanical loading. We used solute diffusion and energy metabolism models to explore how mandibular size affects TMJ disc oxygen and glucose availability, lactate accumulation, and ATP production (30). We simulated the effect of TMJ disc compression during static biting on the nutrient environment of the disc. This involved recreating localized solute exchange reduction on the contact area and incorporating mechanical strain–dependent solute diffusion in the disc’s loading volume. Movement of solutes within the disc were governed by Fick’s second law. Oxygen and glucose consumption rates and ATP production were calculated according to stoichiometry of intracellular energy metabolic reactions (31) (Figure 6A).

Individuals with smaller mandibles and larger joint forces had worse TMJ disc oxygen and glucose availability, more lactate accumulation, and less ATP production; 3D mandibular length was most strongly correlated with these mechanobiological indicators compared with other mandibular size indicators (Supplemental Figure 6; Supplemental Table 7). Large joint forces could cause a large TMJ disc–condyle contact area and increased mechanical strain, slowing TMJ disc nutrient and metabolism waste transport (Supplemental Figure 7). Our solute diffusion and metabolism model provides a mechanistic explanation for why degenerative changes are more likely to happen in people with small mandibles (Figure 6B).

### Role of condyle shape and size in determining joint contact area and energy density.

**Figure 7:**
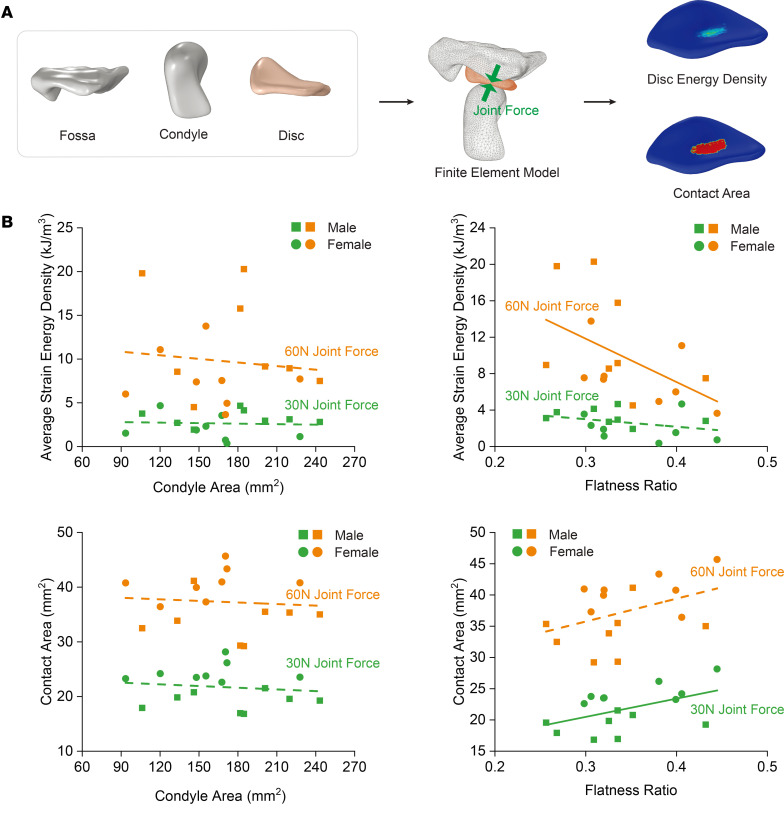
Evaluation of contact behavior and tissue response in the TMJ using finite element mechanics models. (**A**) The models encompass the TMJ condyle, fossa, and disc and provide insights into strain energy density, the energy deposited in the TMJ disc that can lead to tissue fatigue and damage. To assess contact behavior independent of joint force, constant joint force loads of 30 N and 60 N were applied. A fixed boundary condition was set for the fossa, with the joint force exerted on the condyle. The resulting TMJ disc strain energy density and contact area are depicted. (**B**) Analysis suggests a mild influence of condyle shape on determining energy density and contact area (*n* = 16, 8 males and 8 females). Average strain energy density versus condyle area (30 N: *P* = 0.816, R² = 0.0040; 60 N: *P* = 0.671, R² = 0.0133). Average strain energy density versus flatness ratio (30 N: *P* = 0.192, R² = 0.1182; 60 N: *P* = 0.041, R² = 0.2668). Contact area versus condyle area (30 N: *P* = 0.753, R² = 0.0073; 60 N: *P* = 0.615, R² = 0.0186). Contact area versus flatness ratio (30N: *P* = 0.048, R² = 0.2509; 60N: *P* = 0.101, R² = 0.1800). Solid lines represent curve fittings where the differences are statistically significant (*P* < 0.05) and dashed lines represent curve fittings where the differences are not statistically significant (*P* ≥ 0.05). While condyle size dictates the potential contact area capacity, actual contact is influenced more by shape. Even under a large joint force (60 N), less than half of the condyle area engaged in contact. A steeper condylar shape ensured improved joint congruency, distributing the load across a broader area. Interestingly, the shape of the TMJ condyle was found to be independent of mandibular size (*r* < 0.1), indicating that both mandibular size and condylar shape function as distinct morphological risk factors for TMJ disorders.

TMJ finite element mechanics models were used to evaluate contact behavior and the tissue response. The models include the TMJ condyle, fossa, disc, and output strain energy density, which is the energy deposited in the TMJ disc that can cause tissue fatigue and damage. We previously showed that joint force is influential, so to study contact behavior independent of joint force, we used constant joint force loads of 30 N and 60 N. We applied a fixed boundary condition to the fossa and exerted the joint force on the condyle. TMJ disc strain energy density and contact area were recorded (Figure 7A).

Flat TMJ condyle shape, but not condyle size, was associated with larger energy density and small contact area. Morphological indicators such as TMJ condyle size and shape determine contact area with condyle size representing the contact area capacity. However, our results showed that even with large joint force (60 N), less than 50% of the condyle area made contact. Instead, condyle shape played a major role; steep condylar shape allowed for better joint congruency and distributed the load over a larger area. TMJ condyle shape was independent of mandibular size (*r* < 0.1), suggesting that both mandibular size and condylar shape are independently important TMJ disorder morphological risk factors (Figure 7B).

## Discussion

Traditional musculoskeletal research methodologies, which often rely on observation-based hypotheses, have proven effective for addressing single-factor clinical challenges. However, the human capacity to discern patterns within vast amounts of complex data is inherently limited; we require tools to decipher the intricate patterns within large and complex data sets. The availability of large data sets in contemporary research presents an opportunity to employ data-driven approaches for deeper insights, and deep learning has emerged as a potent tool. Yet, the black-box nature and other intrinsic limitations of traditional machine learning often restricts its ability to elucidate the underlying mechanisms driving detected patterns. Our adoption of explainable deep learning improves upon traditional machine learning by identifying the features based on which the model made its prediction, providing improved model insights and enhancing the ability to further probe deep learning outputs.

Beyond recognizing patterns and relationships within data, it is imperative to uncover the underlying mechanisms behind potential risk factors and comprehend their effect on biological tissues. In musculoskeletal disorder research, multiscale biomechanical modeling has been recognized as an effective method to investigate mechanisms of pathology. However, such modeling requires a clear understanding of relevant potential risk factors; as previously noted, traditional methods are often driven by an incomplete representation of related risk factors and as such fall short in providing a holistic view of disorder mechanisms. To bridge this gap, our study introduces a pioneering hybrid approach that synergizes the complementary capabilities of 3D explainable deep learning and multiscale biomechanical modeling. This innovative methodology not only facilitates a comprehensive understanding of complex conditions, such as TMJ disorders, but also delivers mechanistic insights into underlying tissue responses. When adeptly integrated, explainable machine learning and multiscale biomechanical modeling hold immense promise for tackling multifactorial conditions in biomedical research.

This hybrid methodological approach can be utilized to better understand a wide array of musculoskeletal disorders and diseases; here, we have used TMJ disorders as an example of the approach’s utility. TMJ disorders, like many multifactorial musculoskeletal conditions, present considerable clinical challenges due to a limited understanding of their complex origins and multifactorial risk factors. Present treatments primarily offer temporary symptom relief, often through pharmacological pain management, instead of addressing the root causes (32–34). Consequently, in light of the ongoing opioid crisis, there is a pressing need for improved methods to better understand TMJ disorder etiology and comprehensively assess risk factors. The TMJ disorder challenge underscores a broader challenge in biomedical research: the need for innovative methodologies that can unravel the intricate interplay of factors in complex conditions and provide reliable mechanistic explanations. Previous research has leveraged 2D deep learning algorithms for improved diagnostic prediction of TMJ disorder–related conditions (21, 35), which demonstrated the utility of deep learning in objectively interpreting complex TMJ morphology. However, prior limitations reduced clinical applicability, accuracy, and translational confidence of previous models: (a) lack of complete 3D representation of TMJ morphology, (b) black-box output without insight into specific morphological regions influencing TMJ disorder risk, and (c) exclusive reliance on deep learning, which does not elucidate the mechanical and functional relevance of morphological features. This study directly addresses these limitations, illustrating the value of combining explainable deep learning and multiscale biomechanical modeling in TMJ research.

This investigation employed 3D explainable deep learning within a clinically and biomechanically relevant context, marking a considerable step forward from traditional assessment techniques for identifying TMJ disorder morphological risk factors. Traditional approaches have been hindered by intrinsic challenges tied to human inference and 2D imaging, such as the confounding effects of scaling and distortion, inconsistent distribution of morphological features across individuals, and the human limitation in recognizing and memorizing intricate patterns (36). On the other hand, 3D explainable deep learning offered a systematic and comprehensive means to grasp the morphological risk factors associated with TMJ disorders. The participant-specific combinations of condylar, ramus, and/or chin regions as areas of interest found in each participant’s saliency map underscore the multifactorial and intricate nature of TMJ disorder morphological risk factors. This complexity extends beyond the simplistic notion of a single most important region, highlighting the interconnected relevance of multiple morphological features. In fact, it is this complexity that validates our adoption of explainable deep learning. The notable findings of our study, specifically the correlation of mandibular size and condylar shape with TMJ disorder risk, highlight the value of integrating advanced machine learning and musculoskeletal modeling for precise identification and interpretation of intricate morphological traits.

Mechanistic understanding of the relevance of identified TMJ disorder morphological risk factors to the TMJ internal environment is essential. Using a hybrid approach combining deep learning and multiscale biomechanical modeling, the current study determined that smaller mandibular size increases TMJ internal reaction force by altering the moment arm ratio between muscle force and bite force. This results in worse TMJ disc oxygen and glucose availability, more lactate accumulation, and less ATP production by increasing the contact area between the TMJ disc and condyle. This finding suggests that women and patients with class II skeletal malocclusion, who typically have smaller mandibular sizes, may be more likely to develop TMJ disorders (23–25) due to larger joint forces and inadequate nutrient availability and ATP production.

Previous studies have identified morphometric measurements such as reduced ramus height, narrower ramus width, and smaller condyle volume in patients with TMJ disorder patents compared with healthy controls using traditional approaches (9, 10). Our explainable deep learning model also identified these features, which confirmed the robustness of our methodology. However, previous studies failed to reveal the true risk factor, mainly because they studied each piece of local information separately. Our study adopted a holistic approach and evaluated the risk factors all together. Our results showed that the true risk factor is overall mandible size, which is closely correlated with the moment arm ratio between muscle force and bite force. Grasping this underlying mechanism can transition these findings into practical applications. For instance, orthognathic surgeries can target alteration of the moment arm ratio, which, in turn, impacts joint loading. This offers a foundational rationale for addressing TMJ issues through orthognathic surgical procedures. Some morphological patterns, such as a smaller condyle size, might be observed in the TMJ disorder group but might not induce substantial biomechanical changes in the TMJ. As such, these patterns should be considered more as indicators of TMJ disorders rather than direct risk factors. Additionally, we discovered that a flatter condylar shape can increase TMJ disc strain energy density by affecting the joint contact area. Given that degenerative changes caused by large strain energy density can further flatten the condyle, early intervention is crucial. Without timely action, a vicious cycle might ensue, ultimately leading to TMJ disorders.

Our results have provided mechanistic understanding as to the clinical and biomechanical relevance of the risk factors identified by the deep learning model. This additional context critically supports TMJ disorder etiological understanding, provides greater translational confidence in applying deep learning to improve TMJ disorder patient care, and enables informed risk analysis and development of targeted treatment modalities. Of note, improved context and mechanistic explanation provided by the proposed hybrid approach also enable deep learning application with smaller data sets, improving clinical accessibility of this powerful methodology compared with traditional machine learning approaches.

The broader implications of this study extend beyond TMJ disorders, signaling a paradigm shift in biomedical research. Morphology drives joint mechanics, and joint mechanics drive mechanobiological responses; understanding each of these pieces of the mechanistic chain is critical to improve the etiological understanding of musculoskeletal disorders and optimize care for affected patients. By synergizing explainable machine learning with multiscale biomechanical modeling, we’ve illuminated specific morphological risk factors and their biomechanical implications in TMJ disorders. With this hybrid approach, the interpretive context provided by biomechanical models describes the mechanobiological relevance of machine learning model outputs, increasing methodological accessibility with smaller and more widely available clinical data sets. Indeed, developed models could serve as potent screening tools, considering multifaceted risk factors simultaneously. Applying this methodology with larger data sets amassed from collaborative efforts across multiple clinical sites would further improve the robustness and translational strength of machine learning outputs. This approach not only addresses the root etiology of musculoskeletal conditions but also enables development of tailored and effective interventions. In an era when biomedical research can be supported with complex disease data sets, such pioneering strategies utilizing available data are essential for enhancing patient outcomes and addressing complex public health challenges.

While our model proved accurate in identifying patients with TMJ disorder, limited sample sizes for training and validation presented a challenge. This might have skewed the model toward overfitting and led to the possibility of missing morphological indicators not well represented in our data set; to mitigate these limitations, we used random flipping and random erasing to prevent overfitting. Another limitation is that the age distribution is different within the data set for the neural network and the data set for multiscale biomechanical models; the neural network data set predominantly featured individuals averaging 34.6 years, aligning with the typical prevalence age for TMJ disorders, while the biomechanical modeling data set consisted mainly of older individuals, averaging 74.2 years, due to the greater availability of cadavers from this age group. To counteract potential biases from these age differences, we implemented methodological safeguards such as using cadavers without TMJ symptoms and separately applying data sets to their respective study areas — neural network training and biomechanical modeling — to minimize the influence of age on our results. This study focused on morphological risk factors and thus assumed nonparticipant-specific parameters across all participants. However, crucial factors such as participant-specific kinematics data, musculoskeletal behavior, local biological environment, and material properties have the potential to affect modeling outcomes. Future research will collect and analyze a substantial quantity of multimodality data to address the aforementioned factors. The identification of a more comprehensive set of risk factors and their interactions could help to further understand TMJ disorder etiology for early diagnosis and optimized treatment.

In conclusion, our hybrid approach combining 3D explainable deep learning and multiscale biomechanical modeling offers a robust methodology for systematically identifying risk factors and providing mechanistic explanations in multifactorial musculoskeletal conditions. While this study focused on TMJ disorders, revealing the implications of small mandibular size and flat condylar shape on TMJ biomechanics, the broader impact lies in the approach’s potential applicability. Deep learning, as an analytical tool enhanced to provide explainable outputs, has shown immense promise in offering systematic and objective insights into complex data sets. However, to truly harness its potential in patient care, we need to understand underlying mechanisms to boost translational confidence; our methodology provides this additional necessary context using multiscale biomechanical modeling. Beyond TMJ disorders, this innovative approach can be instrumental in understanding other multifactorial musculoskeletal pathologies, including knee osteoarthritis, spine disc degenerative diseases, and associated pain conditions, underscoring its versatility and broad relevance in biomedical research.

## Methods

*Sex as a biological variable*. Our study incorporated data from both male and female individuals, utilized in explainable machine learning and multiscale biomechanical modeling. Although sex was not considered as an independent variable in the construction of our explainable deep learning models, due to the focus on morphological risk factors, we have differentiated between male and female participants in the presentation of our multiscale biomechanical results. This differentiation was implemented by labeling male and female participants in different shapes for mechanistic understanding.

*Explainable machine learning model*. CBCT images were obtained from 52 patients with TMJ disorder exhibiting disc displacement and 52 healthy controls without functional loss. Patient data were examined by a calibrated examiner under the standardized DC-TMJ disorders Axis I and II protocols (37) and collected under institutional approval. All patient data were fully anonymized to ensure privacy. 3D mandible geometries were extracted from CBCT images and segmented into STL format using Amira (Thermo Fisher Scientific). The geometries were centered and voxelized, resulting in a resolution of 60 × 45 × 45, with each voxel representing a 2.5mm^3^ cube in real space. The voxelized sample was represented as a 3D binary matrix, with ‘1’ indicating regions occupied by the mandible and ‘0’ indicating empty regions. Data augmentation was employed to improve the diversity of training data and mitigate overfitting. Random flipping and random erasing techniques were applied, taking into consideration the symmetry of mandible geometries and the characteristics of voxelized models.

A 3D convolutional neural network was employed for participant classification (Figure 2A). The model is comprised of 3 3D convolutional layers for feature extraction, global average pooling, and 2 fully connected layers with 128 hidden neurons. The activation function used between layers was ReLU (rectified linear unit). To prevent overfitting, a dropout operation with a 0.3 probability was used between the fully connected layers. The output of the model was a scalar value for each voxelization matrix, which was then used to perform classification through the sigmoid operator. The model parameters were optimized by minimizing the binary cross-entropy loss. To maintain classification accuracy while managing model complexity, the model was designed to be relatively shallow, preserving position information during saliency map generation by gradient backward propagation.

Data were split into training (40 TMJ disorder, 40 healthy samples) and validation (12 TMJ disorder, 12 healthy samples) sets. The training set was processed with random data augmentation at each training iteration. A dropout operation with a 0.3 probability was implemented between the 2 fully connected layers during training. The Adam optimizer (38) was used with a learning rate of 0.001 for parameter optimization. Training was performed on a Nvidia V100 GPU-equipped machine, taking approximately 3 hours with 2,000 iterations and a batch size of 80 samples. The model achieved an accuracy of 83.33% when evaluated on the validation set.

The efficacy of our deep learning model was further gauged with a ROC analysis (26) by calculating the AUC ROC following a 10-fold cross-validation procedure. This measure, routinely used to assess the accuracy of binary classification models, provides a value ranging between 0.5 and 1.0. Here, a score of 0.5 implies no better performance than random chance, whereas a perfect score of 1.0 indicates flawless classification. Optimal model sensitivity and specificity were also identified in the ROC analysis, representing the true positive and true negative rates, respectively. A perfect sensitivity score of 1.0 would indicate the ability to correctly identify all patients with TMJ disorder as having a TMJ disorder, whereas a perfect specificity score of 1.0 would indicate the ability to correctly identify all healthy controls as being without TMJ disorder.

To visualize and understand the morphological characteristics of TMJ disorders that contributed most to the classification decisions of our model, we utilized a method known as Grad-CAM (27) for generating saliency maps. In brief, the Grad-CAM approach is designed to highlight the critical regions within the input data that have a substantial influence on the model’s output prediction. This is achieved by calculating the gradients of the output category with respect to the feature maps of a specific convolution layer. These gradients are then globally average-pooled to obtain importance weights for each feature map. The final saliency map, or Grad-CAM heatmap, is created by taking a weighted combination of the feature maps guided by these importance weights.

In the context of our binary classification model, we adjusted the standard Grad-CAM method to compute gradients directly through the model’s output, rather than with respect to a specific output category. This modified approach allowed us to quantify how changes in the activation of different regions within the 3D image would affect the overall classification output of the model. To generate the final saliency maps, we rescaled the weighted combination of activation maps and projected them back onto the original input voxel space. This resulted in saliency maps that highlighted the regions within the input CBCT images that were of most importance for the classification decisions of our model. These maps provide valuable insights into the model’s decision-making process and help make it more understandable and interpretable. Detailed mathematical derivations and equations supporting our methodology are provided in Supplemental Note 1. The neural network and Grad-CAM algorithm were implemented using PyTorch framework.

*Inverse dynamic musculoskeletal model*. The heads of 8 male and 8 female cadavers were scanned using a CBCT scanner (Planmeca3D Max, Planmeca USA) and a 7T MRI scanner (BioSpec 70/30 USR; Bruker Corp.). The voxel dimensions for the CBCT scanner were set at 0.2 × 0.2 × 0.2 mm^3^, while the MRI in-plane resolution was set at 0.234 mm × 0.234 mm with a slice thickness of 0.5 mm. After the scans were completed, dissection was performed to record muscle attachment sites using a camera-based 3D location recording system.

EMG activities of bilateral temporalis and masseter muscles were recorded from a representative male and female individual using surface electrodes (Biometrics) during a static biting task. Concurrently, bite forces were captured via a calibrated force sensor. Participants were asked to perform static biting tasks with gradually increasing bite force magnitude, from 10 N to 70 N. During these tasks, the kinematics of the mandibular system were recorded using a camera-based motion capture system (Supplemental Figure 4). This extensive data allowed us to develop a model capable of accurately simulating the biomechanical behavior of the TMJ across a spectrum of bite forces, including the 11 N, 30 N, and 60 N we chose in this paper.

A high-pass zero-lag fourth-order recursive Butterworth filter (cut-off frequency: 30 Hz) was utilized to remove the DC offset of the raw EMG signal caused by movement artifacts of the electrodes. The signal was then full wave rectified, normalized with respect to the peak rectified EMG value obtained during the maximum voluntary contraction (MVC) experiment, and filtered using a Butterworth low-pass filter (cut-off frequency: 6 Hz) (39).

Sixteen participant-specific mandibular system musculoskeletal models were constructed using the bone geometry obtained from the CBCT scans and muscle attachment information obtained from dissection. The models were driven by the mandible kinematics, EMGs, and bite forces obtained from the 2 reference participants: 1 male reference participant and 1 female reference participant. Male cadavers were driven by data from the male reference participant, while female cadavers were driven by data from the female reference participant.

Inverse dynamics–based 3D musculoskeletal models of the human mandible were developed using our previously described approach (28). Moment of inertia used in the model can be found in Supplemental Table 3. The relative movement of the lower jaw with respect to the skull during maximal jaw open-close motion was calculated using the coordinate transformation method.

The musculoskeletal model of the mandible (28) was equipped with 26 muscle actuators, including 5 distributed forces for the temporalis and masseter muscles and 3 centroid-to-centroid forces for superior/inferior lateral and medial pterygoid muscles on each side of the cephalad. Muscle parameters are summarized in Supplemental Table 4. The initial estimation of muscle forces was made by inputting the low-pass filtered EMGs and instant muscle lengths/velocities into the Hill-type muscle model (Supplemental Note 2; Supplemental Figure 3) considering muscle force-length, force-velocity, and passive elastic force-length relationships (40).

The resultant forces and moments at the TMJ were calculated using principles of inverse dynamics. To address the redundancy issue of shared muscle forces concerning the mandible force equilibrium equations, an optimization program was used to minimize the difference between the resultant mandible moment calculated from inverse dynamics and the resultant mandible moment estimated from the Hill-model. Experimental model validation can be found in our previous publication (28).

*Solute diffusion and metabolism model*. The nutrient environment within the TMJ disc was calculated for the same 16 participants using solute diffusion and energy metabolism models (30). This analysis generated 3D concentration gradient profiles of oxygen, glucose, lactate, and ATP production.

Solute gradients within the disc were calculated using Fick’s second law, a principle that describes solute diffusion processes:


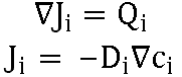


where J_i_ represents the flux of a given solute species, Q_i_ the metabolic reaction for that species, D_i_ the diffusion coefficient, and c_i_ the concentration. The metabolic reactions were defined based on the stoichiometry of intracellular energy metabolic reactions in Supplemental Note 3.

In our first stationary study step, diffusion coefficients for the nutrient solutes (glucose, oxygen, and lactate) were held as constant values derived from literature-reported measurements (30). During the second transient study step, we quantified the disruption of solute gradients of the TMJ disc in response to 2-hour static biting. The effect of TMJ disc compression under static biting on the disc nutrient environment was simulated by mimicking the attenuated localized solute exchange through the condyle-disc contact area and considering mechanical strain-dependent solute diffusion in the loading volume of the disc. Definitions of condyle-disc contact area and disc loading volume are based on normal stress as described in Supplemental Note 4. The mechanical strain-dependent diffusion coefficients were determined using the strain values obtained from the finite element mechanics models, as detailed in Supplemental Note 5.

We quantified changes in nutrient availability and accumulation of waste metabolites. These were presented as a normalized percentage change in the mass of a specific molecule (denoted by i), where i can represent oxygen, glucose, lactate, or ATP, within the entire volume of the disc. These changes were derived from the 2-hour loaded configuration compared with the initial unloaded configuration:


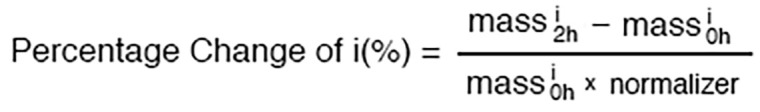


where normalizer is determined by finding the maximum ratio of initial mass of i in the loading volume to the initial mass of i across the whole disc, across all participants studied; mass^i^_0h_ denotes the mass of i across the whole disc, mass^i^_2h_ denotes the mass of i across the whole disc after 2 hours under load. Other modeling parameters, including those measured experimentally, are described in Supplemental Table 5. The validity of this approach is experimentally tested by our previous work, wherein in vitro cellular metabolic measurements corresponded well with the outcomes predicted by our model (30).

### Finite element mechanics model.

Finite element models were built to provide deeper understanding of the complex TMJ disc biomechanical behavior under various static biting conditions.

Employing the data from the heads of the same 16 cadavers used in the Inverse Dynamic Musculoskeletal Model, finite element models of the TMJ were developed. The specimens were subjected to CBCT and 7T MRI scans. Utilizing COMSOL Multiphysics software, these models were constructed with the aim of evaluating the biomechanical responses within the TMJ articular disc.

Segmentations of the TMJ fossa, condyle, and TMJ disc were extracted from CBCT and MRI scans using Amira software (Thermo Fisher Scientific). The finite element model was built to include 5 material components: the condylar head, temporal fossa, articular disc, condylar cartilage, and temporal fossa cartilage. Contact pairs were defined between both cartilages and disc. Linearly elastic material properties were assigned to these components with reference values provided in Supplemental Table 6. A fixed boundary condition was applied to the temporal fossa and the condyle was defined as movable. Joint force was applied to the articular disc through the TMJ condyle to simulate the disc biomechanical response under static biting.

The elastic strain energy density was recorded to understand the tissue mechanical behavior and fatigue risk. Equations used to calculate these measures are provided in Supplemental Note 6.

### Statistics.

This study utilized a mixed effects model, implemented through the ‘mixedlm’ function of the ‘statsmodels’ Python library (41), to discern the differences between the TMJ disorders and healthy groups. In this model, the TMJ disorders or healthy group membership was a fixed effect, representing the primary interest, and the individual was included as a random effect to accommodate correlation between measurements from the left and right sides of the same participant. The model was estimated using the Restricted Maximum Likelihood (REML) method, a preferred estimation technique for mixed models, as it provides unbiased estimates of variance and covariance parameters. No adjustments were made to the reported *P* values. Selected results are reported in the manuscript, with additional details provided in Supplemental Table 2.

A PCA was performed using MATLAB’s built-in ‘pca’ function and was subsequently verified with OriginLab Pro. Given that the measurements were all in the same unit but with different means, data were normalized using the ‘zscore’ function prior to PCA, ensuring that each parameter contributed equally to the principal components, thus preventing any single measure from dominating due to scale. The correlation between the original variables and the principal components was calculated to interpret the importance of the components.

The linear regression analysis was conducted using OriginLab Pro to examine the relationship between morphological indicators and the results of multiscale biomechanical modeling simulations. The significance level was set at α = 0.05 for the analysis. The *P* values and R^2^ values were calculated and are reported to assess the null hypothesis that the slope is zero and to quantify the proportion of the variance in the simulation results that is predictable from the morphological indicators.

### Study approval.

This study is the under the Medical University of South Carolina IRB approval of Pro00077484 and Pro00066351.

### Data availability.

The data sets supporting the conclusions of this article are divided into 2 main categories: an explainable deep learning data set and a multiscale biomechanical modeling data set. The explainable deep learning data set contains comprehensive information for each participant, including the actual medical diagnosis, machine learning predictions, identified regions of interest based on Grad-CAM, sigmoid activation values, and detailed morphological measurements. The multiscale biomechanical modeling data set contains detailed modeling results for each participant, including joint forces, average disc strain energy density, disc contact area, glucose and oxygen availability, lactate accumulation, and ATP production. All these data are in the Supplemental Materials or Supporting Data Values files published along with this paper.

Due to their substantial size, the CBCT data, finite element models, and analytic code used in this study are not included in this article. Interested parties can obtain these materials by contacting the corresponding authors via email.

## Author contributions

SS, PX, YW, and HY designed the research study. SS, PX, NB, FA, MBW, AM, XS, BS, YW, and HY conducted experiments. SS, NB, CNH, EHS, YW, and HY analyzed data. SS, PX, NB, YW, and HY contributed materials and/or analysis tools. SS, CNH, EHS, JSL, YW, and HY wrote the manuscript. The order of cofirst authorship was determined based on individual contributions. SS is recognized as the first cofirst author for his substantial role in the research study. He was instrumental in designing the study and coled the entire project, including both explainable deep learning and multiscale biomechanical modeling. PX is also acknowledged as a cofirst author. His primary contribution was designing the study, with a focused involvement in the explainable deep learning aspect, demonstrating his specialized skills in this area. NB made a substantial contribution by coleading the multiscale modeling efforts. Based on the importance and amount of contribution of each first coauthor, we decided the order.

## Supplementary Material

Supplemental data

Supporting data values

## Version 1 07/11/2024

In-Press Preview

## Version 2 08/22/2024

Electronic publication
