# Supplementary material for: Explainable deep learning and biomechanical modeling for TMJ disorder morphological risk factors
Source: JCI Insight. 2024 Jul 11;9(16):e178578. doi: 10.1172/jci.insight.178578 (PMC11343598; doi:10.1172/jci.insight.178578)
Supplement: Supplemental data [file jciinsight-9-178578-s096.pdf]

SUPPLEMENTARY INFORMATION

Explainable Deep Learning and Biomechanical Modeling for TMJ Disorder Morphological Risk Factors

Authors:

Shuchun Sun, Pei Xu, Nathan Buchweitz, Cherice N. Hill, Farhad Ahmadi, Marshall B. Wilson, Angela Mei, Xin She, Benedikt Sagl, Elizabeth H. Slate, Janice S. Lee, Yongren Wu, Hai Yao

Table of Contents

Supplementary Note 1: Grad-CAM algorithm

Supplementary Note 2: Hill-type Muscle Model

Supplementary Note 3: Stoichiometry of Intracellular Energy Metabolic Reactions

Supplementary Note 4: TMJ Disc Loading Volume and Contact Area Determination

Supplementary Note 5: Mechanical Strain-Dependent Solute Diffusivities

Supplementary Note 6: Definition of Strain Energy Density

Supplementary Table 1. Explainable Machine Learning Results Summary

Supplementary Table 2. Statistical Analysis Results for Morphological Measurements

Supplementary Table 3. Moment of Inertia in Musculoskeletal Model

Supplementary Table 4. Muscle Parameters in Musculoskeletal Model

Supplementary Table 5. Model Parameters in Solute Diffusion and Metabolism Model

Supplementary Table 6. Model Parameters in Finite Element Model

Supplementary Table 7. P-value and R^2 for Supplementary Figure 6

Supplementary Figure 1: Grad-CAM and Grad-CAM++ Saliency Map Comparison

Supplementary Figure 2: Principal Component Analysis Biplot

Supplementary Figure 3: Hill-style Muscle Model

Supplementary Figure 4: Setup for Mandibular Motion, EMG, and Bite Force Capture

Supplementary Figure 5: Mechanistic Insights: Mandibular Size and Joint Force

Supplementary Figure 6: 2D Mandibular Size, Ramus With, Ramus Height and Mechanobiology

Supplementary Figure 7: Mechanistic Insights: Mandibular Size and Nutrient Availability

Reference

Supplementary Note 1: Grad-CAM algorithm

Gradient weighted class activation mapping (Grad-CAM) (1) identifies the most influential regions for model prediction by computing the weighted sum of the final activation maps of the convolutional layers, namely, the ReLU layer output before the average pooling in our model. The computation is done by:

$$\text{Grad-CAM}(x_i) = \text{ReLU}\left(\sum_k \alpha_i^k A^k(x_i)\right)$$

where $A^k(x_i) \in \mathbb{R}^{46\times31\times31}$ is the $k-th$ channel output of the final convolutional layer with the activation function $\text{ReLU}(\cdot)$. In the vanilla Grad-CAM, the weight α_i^k is achieved using the gradient $\frac{\partial y_i}{\partial A^k(x_i)}$ with respect to the class-specified one-hot vector y_i. To support our binary classification model, we compute the gradient through the model output directly and use the counterfactual explanation to identify the regions of interest. More specifically, we compute the weights of each activation map contributing to the classification result by

$$\alpha_i^k = -\sum_{l,m,n} \frac{\partial f(x_i|\theta)}{\partial A_{l,m,n}^k(x_i)}$$

where $k = 1, \cdots, 256$ given that the final convolutional layer in our model has 256 channels and $A_{l,m,n}^k(x_i)$ represents an element in the activation map $A^k(x_i)$. These weights evaluate the contributions that regions in the activation maps would make in changing the classification results. We obtain the saliency map, which denotes the importance of each voxel during model inference, by rescaling the weighted activation map $\sum_k \alpha_i^k A^k(x_i)$ and projecting it back to the input voxel space.

Supplementary Note 2: Hill-type Muscle Model

The Hill-type muscle model (2), a commonly adopted representation of skeletal muscle mechanics, incorporates three key components: the contractile element (CE), the series elastic element (SEE), and the parallel elastic element (PEE). The CE symbolizes the active force generation facilitated by actin-myosin cross-bridges during muscle contraction. A demonstration figure of the Hill-type muscle model can be found in **Supplementary Figure 3**. This active force (F_{active}) produced by the CE is a function of muscle length (L), contraction velocity (V), and activation level (a), as given by the equation:

$$F_{\text{active}} = \mathrm{a} * F_{max} * \mathrm{f_L(L)} * \mathrm{f_V(V)}$$

where F_{max} is the maximum isometric force, $\mathrm{f_L(L)}$ is the length-dependent force multiplier, and $\mathrm{f_V(V)}$ is the velocity-dependent force multiplier.

The SEE and PEE represent the elastic characteristics of the muscle-tendon unit and the passive elasticity of the muscle fibers and connective tissues, respectively, when the muscle is extended. Both the SEE and PEE are usually modeled as nonlinear functions of length. The passive force (F_{passive}) generated by the SEE and PEE is given by the equation:

$$F_{\text{passive}} = \mathrm{f_{SEE}(L)} + \mathrm{f_{PEE}(L)}$$

where $\mathrm{f_{SEE}(L)}$ and $\mathrm{f_{PEE}(L)}$ are the length-dependent force multipliers for SEE and PEE, respectively.

Consequently, the total muscle force, as per the Hill-type muscle model, is the sum of the active force from the CE and the passive forces from the SEE and PEE, as described by the equation:

$$F_{total} = F_{active} + F_{passive}$$

This model delivers a comprehensive understanding of muscle function and forms the basis for muscle performance simulations and the development of biomechanics control algorithms.

Supplementary Note 3: Stoichiometry of Intracellular Energy Metabolic Reactions

Our energy metabolic reactions (3, 4) were defined as follows:

$$Q^{oxygen} = -\rho_{cell} * \frac{v_{max}^{oxygen} * c^{oxygen}}{k_m^{oxygen} + c^{oxygen}}$$

$$Q^{glucose} = -\rho_{cell} * \frac{v_{max}^{glucose} * c^{glucose}}{k_m^{glucose} + c^{glucose}}$$

$$Q^{lactate} = -2 * Q^{glucose} + \frac{1}{3} * Q^{oxygen}$$

$$Q^{ATP} = 2 * Q^{glucose} + 6 * Q^{oxygen}$$

Oxygen and glucose consumption rates Q^{oxygen} and $Q^{glucose}$ were defined based on the Michaelis-Menten kinetics model, where the parameter c denotes the concentration, ρ_{cell} denotes the cell density in the healthy TMJ disc, which was reported in our previous study (ρ_{cell}=50 million cells/mL) (5), v_{max} is the maximum cellular nutrient metabolic rate, and k_m is the solute concentration at which half the maximum metabolic rate is achieved. Lactate and ATP production rates $Q^{lactate}$ and Q^{ATP} were derived from the oxygen and glucose consumption rates. Parameter values used in the model are presented in **Supplementary Table 5**.

Supplementary Note 4: TMJ Disc Loading Volume and Contact Area Determination

We defined the TMJ loading volume as a distinct portion within the TMJ disc, selected based on an identified threshold where average normal stresses exhibited a sudden increase, typically around 0.8 MPa. A scalar value, calculated as the trace of the Cauchy stress tensor divided by three $\frac{\sigma_{ii}}{3}$, served as our determinative factor for this volume, providing an invariant estimate of the stress magnitude imposed on a model element. To identify the value denoting notable stress increases, we fine-tuned the threshold range by closely examining the parameter's gradients visualized at varied angles on a slice plot. This specification was essential for our understanding of the model-derived trends associated with strain energy and nutrient availability, as these results were localized to a minor segment of the disc geometry.

The introduction of the contact area was a strategic move to identify regions on the TMJ disc surface engaged with either the condyle or fossa during our solute diffusion and metabolism simulation. This region was determined by assessing the normal stress magnitude on the disc surface and setting a threshold for surface sections as in the process of defining the loading volume. We further validated the threshold value by ensuring the contact area was congruous with locations experiencing a substantial rise in surface stress magnitude under visual examination. This component was vital for facilitating accurate modeling of solute exchange at the disc boundary within our nutrient finite element models. Furthermore, it yielded crucial insights into the contact behavior within the TMJ.

Supplementary Note 5: Mechanical Strain-Dependent Solute Diffusivities

Mechanical strain dependent small solute diffusion coefficients in fibrocartilage tissues follow an empirical constitutive relation (6): D_{tissue}/D_{aqu}=AΦ^2, where D_{tissue} is the diffusivity in tissue, D_{aqu} is the diffusivity in water, Φ is the water content (tissue volume fraction of water) that can be derived from the initial water content Φ_0 and tissue volumetric strain ε: $\Phi = (\Phi_0 + \varepsilon)/(1 + \varepsilon)$ (7). According to our previous solute diffusion studies, A is 0.11 with Φ_0 equal to 0.7 for TMJ disc tissue (4, 8, 9). Thus, the mechanical strain-dependent diffusivities of nutrient solutes (i.e., oxygen, glucose, and lactate) in the TMJ disc followed relationship:

$$D_{tissue} = D_{aqu} \times 0.11 \times \left[\frac{0.7 + \varepsilon}{1 + \varepsilon}\right]^2$$

where $D_{aqu}^{oxygen} = 3 \times 10^{-5}\ cm^2/\text{sec}$, $D_{aqu}^{glucose} = 0.92 \times 10^{-5}\ cm^2/\text{sec}$, $D_{aqu}^{lactate} = 1.28 \times 10^{-5}\ cm^2/\text{sec}$. These diffusion coefficients were obtained from previously published literature (10, 11)

Supplementary Note 6: Definition of Strain Energy Density

Strain energy density is a critical measure in the analysis of mechanical behavior and fatigue risk. The biomechanical and biochemical integrity of the TMJ disc is dependent on the strain energy density within its solid matrix (12). It refers to the amount of energy per unit volume absorbed by the TMJ disc when it is deformed under an applied load. Strain energy density is calculated by integrating the stress-strain curve up to a given strain, effectively representing the area under this curve. It is mathematically defined as (13, 14):

$$W_s = \int_0^{\varepsilon} \sigma : d\varepsilon$$

where W_s is the strain energy, σ the Cauchy (true) stress tensor, and ε the strain tensor. For linearly elastic materials, which were used in our models, this parameter may be analytically computed as follows (15):

$$W_s = \sigma_{ref}\colon\varepsilon + \frac{1}{2}(C\colon\varepsilon)\colon\varepsilon = \frac{1}{2}\left(\sigma_{ref} + \sigma\right)\colon\varepsilon$$

$$(\sigma = \sigma_{ref} + C\colon\varepsilon)$$

where C is the right Cauchy-Green deformation tensor and σ_{ref} denotes any initial stresses in the material in its reference state.

Supplementary Table 1. Explainable Machine Learning Results Summary

ID	Actual Diagnosis	Predicted Diagnosis	Regions of Interest	Sigmoid
1	control	control	left condyle, left ramus, right condyle, right ramus	0.0072
2	control	control	left ramus, right condyle, right ramus, back of chin, left condyle	0.0071
3	control	control	left ramus, right condyle, right ramus	0.0012
4	control	control	left condyle, left ramus, right condyle, right ramus	< 0.0001
5	control	control	left ramus, left condyle, front of chin, right condyle, right ramus	0.0008
6	control	control	left ramus, right condyle, right ramus	0.0071
7	control	control	back of chin, left forward ramus right condyle, right ramus	0.0066
8	control	control	left ramus, right condyle, right ramus, left condyle	0.0218
9	control	control	left ramus, left condyle, right condyle, right ramus, back of chin	0.0239
10	control	control	front of chin, right ramus, right condyle, left ramus, left condyle	0.0242
11	control	control	left ramus, right ramus, right condyle, chin	0.0071
12	control	control	left ramus, left condyle, right condyle, right ramus, chin	0.0001

13	control	control	front of chin, left upper front of ramus, right ramus	0.0123
14	control	control	Left ramus, right ramus, right condyle	0.0084
15	control	control	right ramus, left ramus, back of chin, left condyle	0.0416
16	control	control	Left ramus, left condyle, right condyle, back of chin	0.0003
17	control	control	left condyle, left ramus, right condyle	0.0008
18	control	control	left condyle, left ramus, right condyle, right ramus	0.0089
19	control	control	left condyle, left ramus, right condyle, right ramus, chin	0.0001
20	control	control	left condyle, left ramus, right condyle, right ramus	0.0010
21	control	control	left ramus, right condyle, right ramus, back of teeth	0.0050
22	control	control	left ramus, right condyle, right ramus	0.0001
23	control	control	left ramus, right ramus, right condyle	0.0292
24	control	control	left ramus, right condyle, right ramus, back of teeth	0.0022
25	control	control	left condyle, left upper ramus, right condyle, right upper ramus	0.0084
26	control	control	left condyle, left ramus, left backside under teeth, right condyle, right ramus	0.0009

27	control	control	front of chin, left forward upper ramus, right condyle, right ramus	0.0001
28	control	control	left condyle, left upper ramus, right condyle, right ramus, back of teeth	0.0073
29	control	control	left ramus, left in front of teeth front of chin, right condyle, right ramus, left condyle	0.0001
30	control	control	left ramus, left condyle, chin, right ramus, right condyle	0.0006
31	control	control	Left ramus, left condyle, right ramus, right condyle	0.0060
32	control	control	left ramus, right condyle, left condyle, right ramus	0.0002
33	control	control	left upper ramus, right condyle, back of chin, left condyle	0.0133
34	control	control	front of chin, left condyle, left upper ramus, right condyle	0.0002
35	control	control	left ramus, left condyle, right ramus, right condyle	0.0030
36	control	control	left ramus, left condyle, right ramus, right condyle	0.0005
37	control	control	left forward upper ramus, right condyle, back of teeth	0.0091
38	control	control	back of chin, right condyle, left ramus, right ramus	0.0016
39	control	control	back of chin, left ramus, right condyle	0.0010

40	control	control	left ramus, right condyle, right ramus	0.0042
*41	control	control	front chin, left ramus, right condyle	< 0.0001
*42	control	control	front chin, left ramus, right condyle	0.3846
*43	control	TMJ Disorders	left ramus, right condyle, right ramus	0.9995
*44	control	control	front of chin left ramus	0.0031
*45	control	control	right forward ramus, right teeth and below, chin	< 0.0001
*46	control	control	right ramus, chin	< 0.0001
*47	control	control	right ramus, front chin, left ramus	0.0100
*48	control	control	right upper ramus, front chin	0.0001
*49	control	control	right upper ramus	0.3435
*50	control	control	right ramus	0.2411
*51	control	control	right ramus, chin	0.0006
*52	control	control	right ramus	0.0117
53	TMJ Disorders	TMJ Disorders	Left ramus, left condyle, right condyle, right ramus, chin	0.9995
54	TMJ Disorders	TMJ Disorders	back of chin, left upper ramus, right condyle	0.9963
55	TMJ Disorders	TMJ Disorders	left condyle, right condyle	0.9996
56	TMJ Disorders	TMJ Disorders	front of chin, left ramus, right condyle	0.9938
57	TMJ Disorders	TMJ Disorders	Left ramus, right condyle, right ramus	0.9919
58	TMJ Disorders	TMJ Disorders	left front of upper ramus, right condyle, right ramus, left condyle	0.9930
59	TMJ Disorders	TMJ Disorders	left ramus	0.9999

60	TMJ Disorders	TMJ Disorders	front chin, left condyle, left upper ramus, right condyle, right ramus	0.9977
61	TMJ Disorders	TMJ Disorders	right condyle, right upper ramus, left ramus	0.9990
62	TMJ Disorders	TMJ Disorders	Left ramus, left condyle, back of chin	0.9992
63	TMJ Disorders	TMJ Disorders	right condyle, right ramus, left ramus	0.9977
64	TMJ Disorders	TMJ Disorders	left upper ramus, right ramus, spotting in teeth	0.9946
65	TMJ Disorders	TMJ Disorders	left ramus, back of chin	0.9941
66	TMJ Disorders	TMJ Disorders	left ramus, right condyle, right ramus	0.9957
67	TMJ Disorders	TMJ Disorders	right condyle, right upper ramus, back of chin, left ramus, left condyle	0.9961
68	TMJ Disorders	TMJ Disorders	Back of chin, right condyle, right ramus, left ramus, left condyle	0.9964
69	TMJ Disorders	TMJ Disorders	Left ramus, right condyle, right upper ramus	0.9855
70	TMJ Disorders	TMJ Disorders	back of chin, left forward ramus, right condyle, right ramus	0.9888
71	TMJ Disorders	TMJ Disorders	right condyle, right ramus	0.9998
72	TMJ Disorders	TMJ Disorders	left forward ramus, right condyle, right ramus, back of chin	0.9900
73	TMJ Disorders	TMJ Disorders	left ramus, right condyle, back of chin	0.9982
74	TMJ Disorders	TMJ Disorders	left condyle, left ramus, right condyle, right ramus	0.9991
75	TMJ Disorders	TMJ Disorders	front of chin, left condyle, left upper ramus, right condyle, right ramus	0.9983
76	TMJ Disorders	TMJ Disorders	Left ramus, left condyle, right condyle, right ramus, back of teeth	0.9944

77	TMJ Disorders	TMJ Disorders	left condyle, left ramus, left behind chin along teeth, right condyle	0.9911
78	TMJ Disorders	TMJ Disorders	Left condyle, left ramus, right condyle, right ramus	0.9942
79	TMJ Disorders	TMJ Disorders	Left ramus, back of teeth, right ramus, right condyle	0.9957
80	TMJ Disorders	TMJ Disorders	right ramus, left ramus, right condyle	0.9948
81	TMJ Disorders	TMJ Disorders	left condyle, left ramus, right condyle, right ramus	0.9961
82	TMJ Disorders	TMJ Disorders	left ramus, right condyle, right ramus	0.9853
83	TMJ Disorders	TMJ Disorders	Right condyle, left ramus, right ramus	0.9874
84	TMJ Disorders	TMJ Disorders	left ramus, back of chin, right condyle	0.9972
85	TMJ Disorders	TMJ Disorders	Left ramus, right condyle	0.9994
86	TMJ Disorders	TMJ Disorders	Left ramus, right condyle	0.9968
87	TMJ Disorders	TMJ Disorders	Left ramus, right condyle	0.9991
88	TMJ Disorders	TMJ Disorders	left ramus, back of chin, right condyle	0.8972
89	TMJ Disorders	TMJ Disorders	front of chin, left ramus, left condyle, right condyle, right ramus	0.5744
90	TMJ Disorders	TMJ Disorders	Left ramus, right condyle, right ramus, chin	0.9963
91	TMJ Disorders	TMJ Disorders	Left ramus, chin, right condyle	0.9795
92	TMJ Disorders	TMJ Disorders	left ramus, right condyle, right ramus	0.9963
*93	TMJ Disorders	control	front chin, left upper ramus, right condyle	< 0.0001
*94	TMJ Disorders	TMJ Disorders	back of chin, left ramus, right ramus, right condyle	0.7015

*95	TMJ Disorders	TMJ Disorders	left front of ramus, right condyle, left condyle, front of chin	0.9905
*96	TMJ Disorders	control	left front of ramus, , right condyle, right ramus	0.1663
*97	TMJ Disorders	control	chin, left upper ramus, right condyle	0.0009
*98	TMJ Disorders	TMJ Disorders	left of chin halfway between chin and ramus, left upper ramus	0.9998
*99	TMJ Disorders	TMJ Disorders	Left forward ramus, right ramus, right condyle	0.9980
*100	TMJ Disorders	TMJ Disorders	left ramus, right condyle	0.9933
*101	TMJ Disorders	TMJ Disorders	right ramus, right condyle	0.9968
*102	TMJ Disorders	TMJ Disorders	left condyle, left ramus Right ramus, right condyle, back of teeth	0.9967
*103	TMJ Disorders	TMJ Disorders	left ramus, right condyle	0.9983
*104	TMJ Disorders	TMJ Disorders	Left condyle, left ramus, right condyle, right ramus	0.9928

* Subjects marked with an asterisk are included in the validation set.

Supplementary Table 2. Statistical Analysis Results for Morphological Measurements

Parameter	TMJ Disorders Mean	TMJ Disorders Variance	Healthy Mean	Healthy Variance	P value
3D mandibular Length (mm)	121.16	52.628	124.78	43.616	0.007
2D mandibular Length (mm)	87.46	25.698	89.80	29.783	0.022
Ramus Width (mm)	32.08	9.42	34.36	15.366	0.001

Ramus Height (mm)	56.92	35.252	59.06	28.772	0.047
Major Axis (mm)	18.82	6.052	20.22	7.728	0.005
Minor Axis (mm)	7.45	1.670	8.30	1.431	< 0.001
Condylar Height (mm)	5.69	0.978	6.54	1.042	< 0.001
Condylar Area (mm^2)	142.63	1340.581	167.41	1359.476	< 0.001
Flatness Ratio	0.31	0.003	0.33	0.004	0.046
Major / Minor Axis	2.59	0.257	2.47	0.177	0.136
Height / Minor Axis	0.78	0.024	0.80	0.026	0.444

Supplementary Table 3. Moment of Inertia in Musculoskeletal Model (16)

Moment of inertia	Mass (kg)	I_{xx} ($kg \cdot m^2$)	I_{yy} ($kg \cdot m^2$)	I_{zz} ($kg \cdot m^2$)
Mandible	0.1	0.000093	0.00015	0.00018

Supplementary Table 4. Muscle Parameters in Musculoskeletal Model (17)

Muscles	Optimum fiber length (mm)	Max force (N)
Superior Temporalis	30.7	106.0
Inferior Temporalis	30.7	106.0
Anterior Temporalis	30.7	106.0
Posterior Temporalis	31.7	106.0
Centroid Temporalis	30.7	106.0
Anterior.1 Masseter	21.8	46.5
Lateral.1 Masseter	15.0	46.5
Posterior Masseter	15.0	46.5

Anterior.2 Masseter	22.6	136.4
Lateral.2 Masseter	22.6	136.4
Superior Lateral Pterygoid	21.5	38.0
Inferior Lateral Pterygoid	22.3	112.8
Medial Pterygoid	14.1	240.0

Supplementary Table 5. Model Parameters in Solute Diffusion and Metabolism Model

**Material Properties**	**Glucose**	**Oxygen**	**Lactate**
**Metabolic Rate (5)**: V_{max} (nmol/million cells/hr)	18.0 (0-6% oxygen)	28.7	Predicted from consumption rates of glucose and oxygen (3) (Supplementary Note 3)
**Metabolic Rate (5)**: K_m (mM)	0.8 (0-2.5% oxygen)	0.019	
	1.6 (2.5-5% oxygen)		
	2.3 (5-6% oxygen)		
**Diffusivity**: D^s (10^{-7} cm^2/sec)	Supplementary Note 5		
**Boundary Conditions**			
Non-contact Area (11, 18) (mM)	4.0	0.06	0.9
Condyle-Disc Contact Area (19-21) (mM)	Impermeable ($\mathbf{n}\cdot\mathbf{J}^{glucose} = \mathbf{n}\cdot\mathbf{J}^{lactate} = \mathbf{n}\cdot\mathbf{J}^{oxygen} = 0$)		
**Initial Conditions**			
Unloaded Disc (11, 18) (mM)	4.0	0.06	0.9

Loaded Disc (mM)	The equilibrium nutrient profiles in the unloaded disc

Supplementary Table 6. Model Parameters in Finite Element Model

Material Component	Elasticity Modulus	Poisson's Ratio
Condylar Head (Bone)(22)	13.7 GPa	0.3
Temporal Fossa (Bone)(22)	13.7 GPa	0.3
Articular Disc(22)	30.9 MPa	0.4
Condylar Cartilage(23)	12.2 MPa	0.4
Temporal Fossa Cartilage(23)	12.2 MPa	0.4

Supplementary Table 7. P-value and R^2 for Supplementary Figure 6

Figure	Loading	p-value	R^2
Oxygen Availability VS 2D Mandibular Size	11N	0.008	0.4067
	30N	0.021	0.3260
	60N	0.204	0.1125
Oxygen Availability VS Ramus Width	11N	0.053	0.2427
	30N	0.039	0.2694
	60N	0.067	0.2194
Oxygen Availability VS Ramus Height	11N	0.157	0.1376
	30N	0.039	0.2713
	60N	0.026	0.3075
Glucose Availability VS	11N	0.002	0.5135
	30N	0.009	0.3985

2D Mandibular Size	60N	0.109	0.1729
Glucose Availability VS Ramus Width	11N	0.031	0.2912
	30N	0.028	0.3020
	60N	0.067	0.2204
Glucose Availability VS Ramus Height	11N	0.027	0.3038
	30N	0.010	0.3038
	60N	0.008	0.4100
Lactate Accumulation VS 2D Mandibular Size	11N	0.732	0.0087
	30N	0.345	0.0638
	60N	0.523	0.0297
Lactate Accumulation VS Ramus Width	11N	0.236	0.0988
	30N	0.124	0.1606
	60N	0.223	0.1040
Lactate Accumulation VS Ramus Height	11N	0.108	0.1738
	30N	0.104	0.1774
	60N	0.183	0.1231
APT Production VS 2D Mandibular Size	11N	0.150	0.1422
	30N	0.120	0.1635
	60N	0.275	0.0845
APT Production VS Ramus Width	11N	0.136	0.1517
	30N	0.085	0.1969
	60N	0.130	0.1560
APT Production VS Ramus Height	11N	0.628	0.0172
	30N	0.181	0.1243
	60N	0.138	0.1504

Supplementary Figure 1: Grad-CAM and Grad-CAM++ Saliency Map Comparison

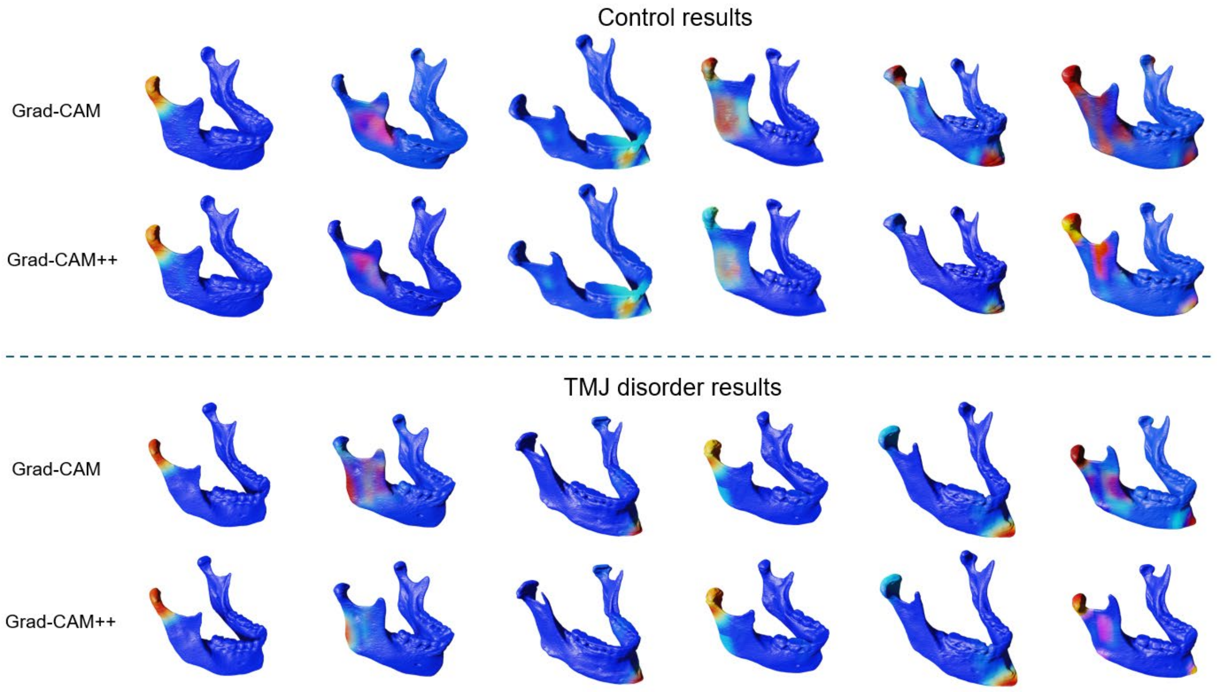

To ensure consistency across different saliency map generation algorithms, we compared the activation maps produced by Grad-CAM and Grad-CAM++. Despite the methodological differences between Grad-CAM, which leverages gradient information, and Grad-CAM++, which additionally considers positive partial derivatives of class scores with respect to convolutional layer outputs, the patterns identified by both algorithms are similar. This similarity underscores the robustness of our analysis and supports the reliability of our findings in highlighting critical regions influencing model predictions.

Supplementary Figure 2: Principal Component Analysis Biplot

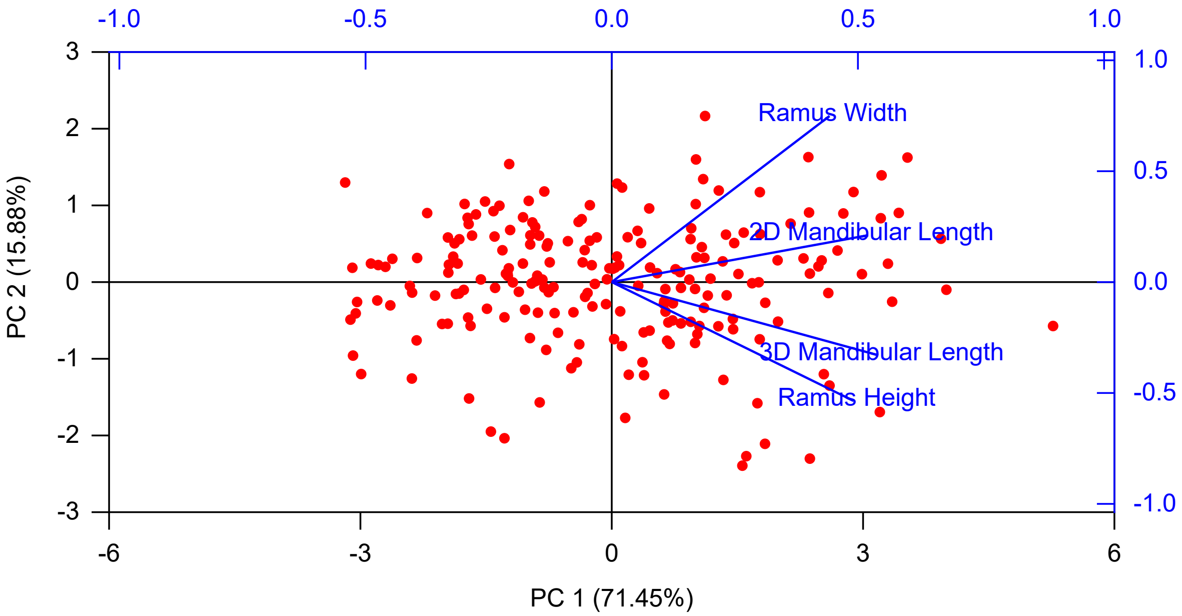

This biplot, constructed from a dataset of n=208 measurements (104 subjects, each with measurements on the left and right side), illustrates the relationship between four morphological measures (3D mandibular length, 2D mandibular length, ramus width, and ramus height) and their contribution to the first principal component (PC1). PC1 accounted for 71.45% of the total variance, with 3D mandibular length contributing the most, showing the highest loading value (0.5413). Other measurements, including 2D mandibular length (0.5202), ramus width (0.4426), and ramus height (0.4905), also had substantial loading values, suggesting that PC1 predominantly encapsulates a 'size' factor in craniofacial morphology and emphasizing the importance of these dimensions in overall facial structure.

Supplementary Figure 3: Hill-style Muscle Model

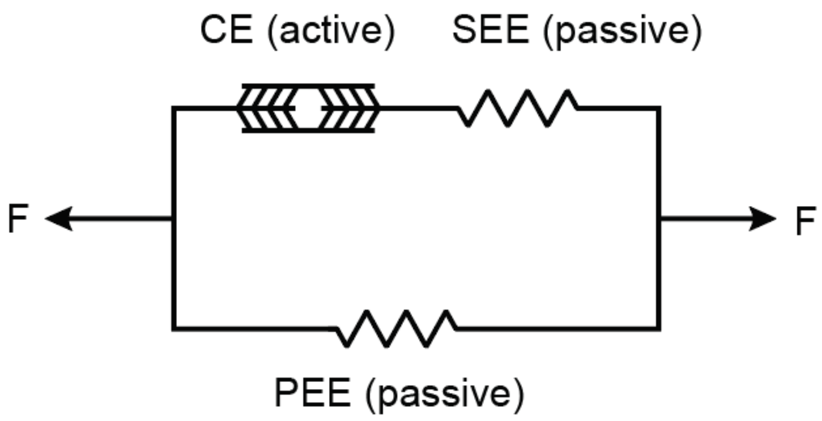

Three key components in Hill-style muscle model: the contractile element (CE), the series elastic element (SEE), and the parallel elastic element (PEE).

Supplementary Figure 4: Setup for Mandibular Motion, EMG, and Bite Force Capture

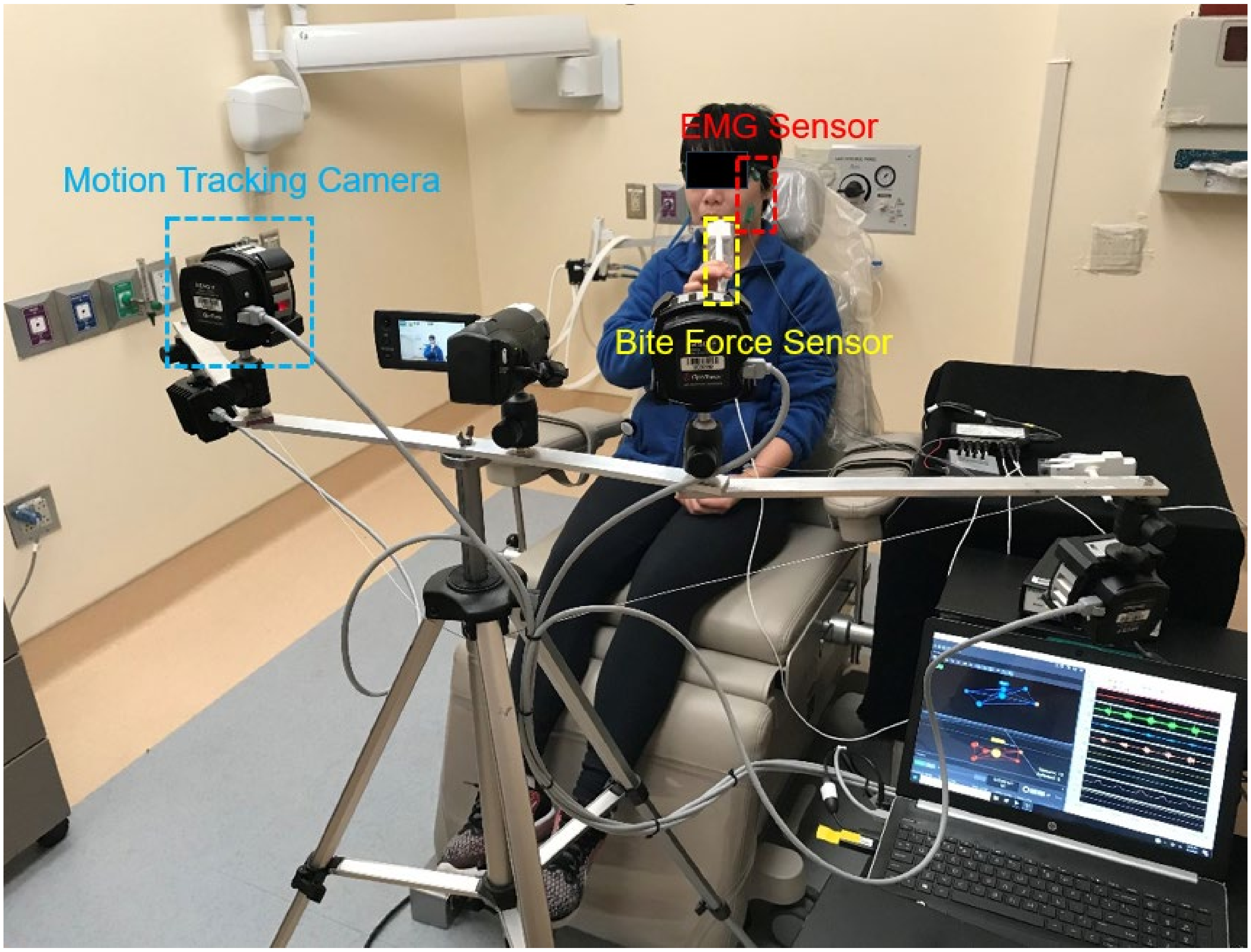

In the examination room, a subject's mandibular motion, electromyograph (EMG), and bite force were captured. The system setup includes a motion tracking system with four cameras, an EMG recording system with sensors attached to the bilateral temporalis and masseter muscles, and a calibrated bite force measurement device. The data collected from this comprehensive setup is integral for constructing subject-specific biomechanical models.

Supplementary Figure 5: Mechanistic Insights: Mandibular Size and Joint Force

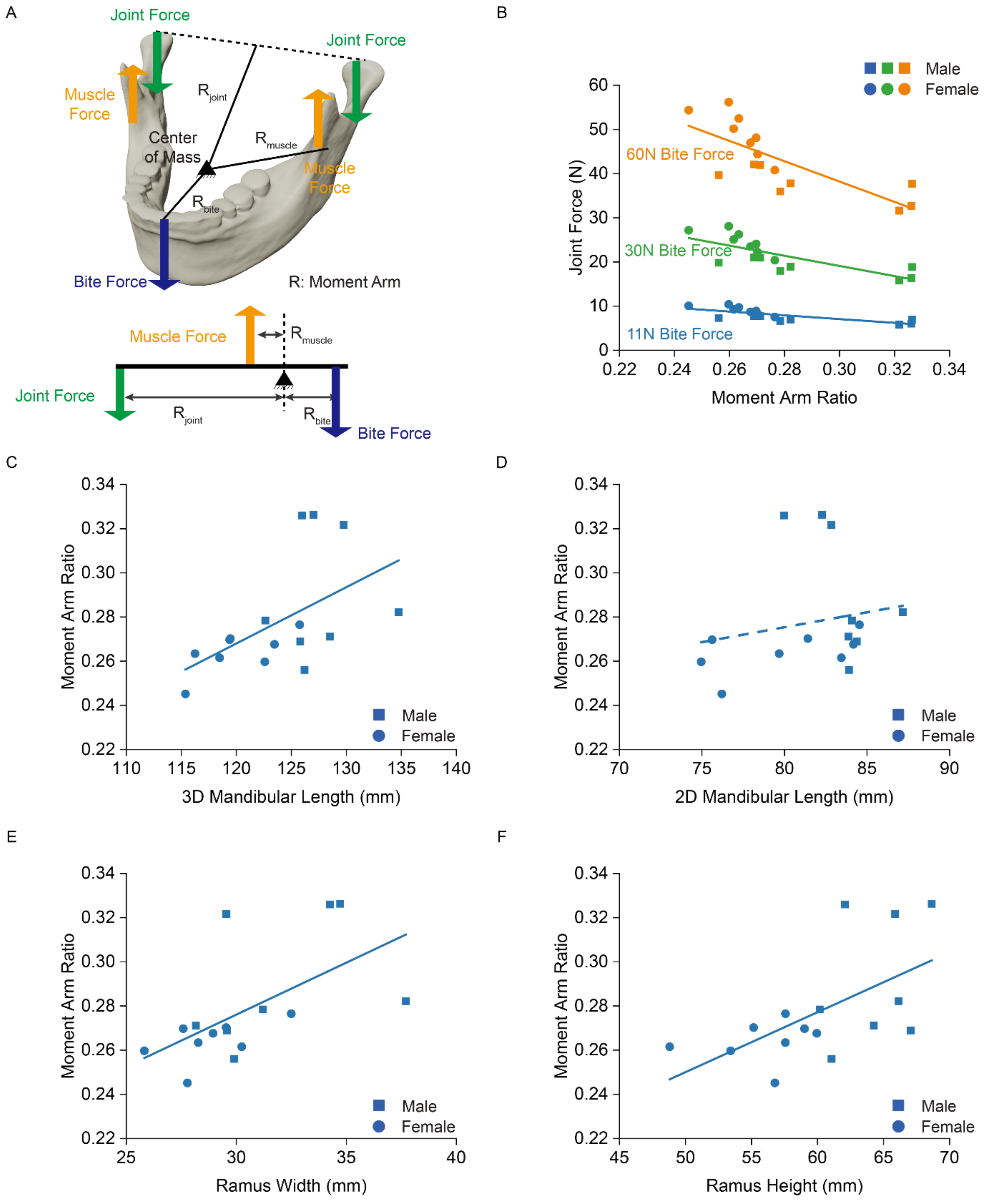

Mechanistic analysis for the relationship between mandibular size and joint force (n=16, 8 males

and 8 females). Solid lines represent curve fittings where the differences are statistically significant ($p < 0.05$), and dashed lines represent curve fittings where the differences are not statistically significant ($p \geqslant 0.05$). (**A**) The mandibular musculoskeletal system can be simplified as a lever with joint force, bite force and muscle force. The moment arm ratio between the bite force and muscle force is what determines the relationship between bite force and muscle force. (**B**) Our simulation data showed that the moment arm ratio is indeed negatively correlated with joint force (11N, 30N, 60N: $p=0.001$, $R^2=0.5848$). (**C**) Relationship between 3D mandibular length and moment arm ratio at 11N bite force ($p=0.034$, $R^2=0.2816$, same relationship at 30N and 60N). (**D**) Relationship between 2D mandibular length and moment arm ratio at 11N bite force ($p=0.472$, $R^2=0.0375$, same relationship at 30N and 60N). (**E**) Relationship between ramus width and moment arm ratio at 11N bite force ($p=0.018$, $R^2=0.3369$, same relationship at 30N and 60N). (**F**) Relationship between ramus height and moment arm ratio at 11N bite force ($p=0.017$, $R^2=0.3442$, same relationship at 30N and 60N).

Supplementary Figure 6: 2D Mandibular Size, Ramus Width, Ramus Height and Mechanobiology

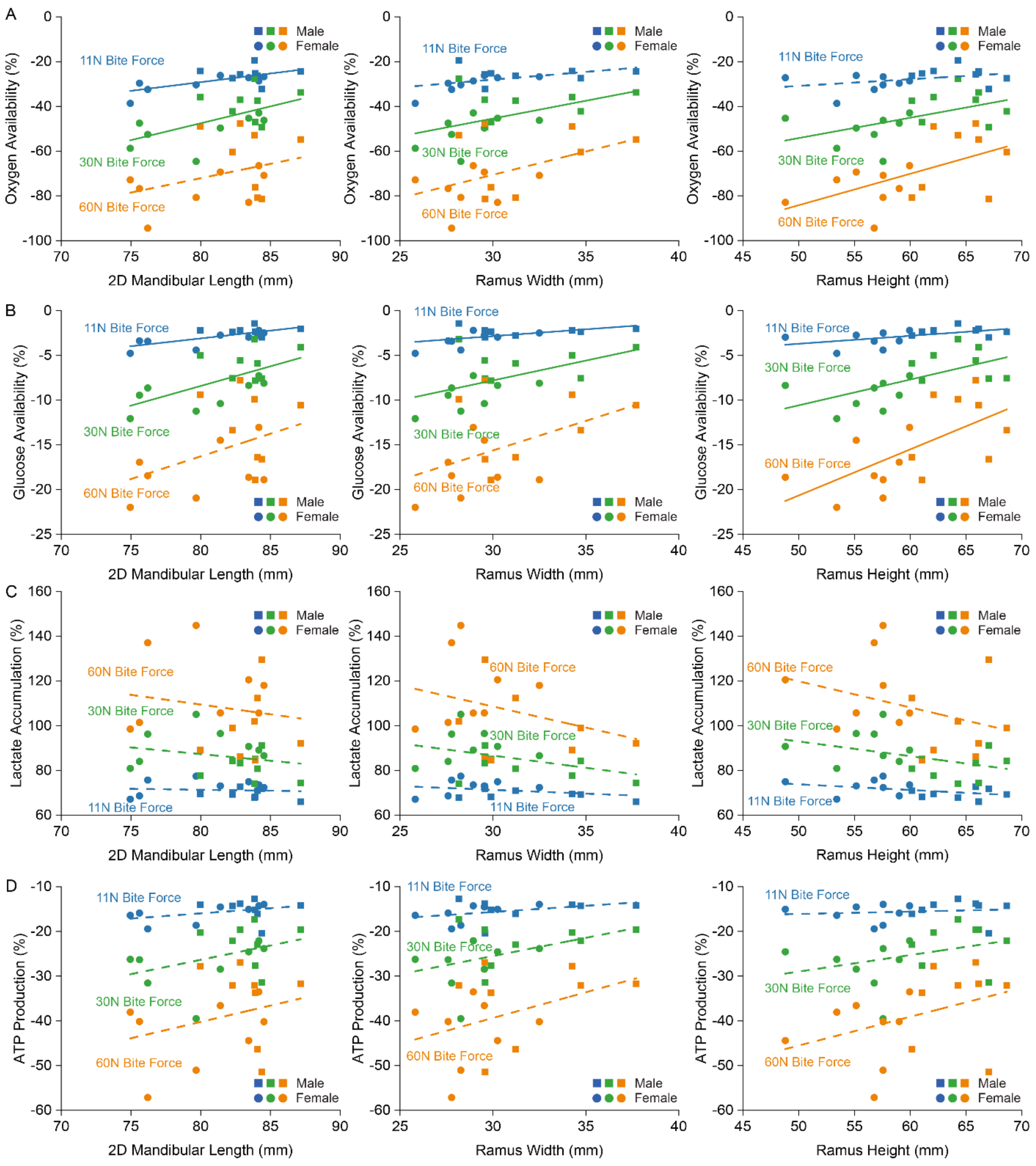

Relationships between 2D mandibular size, ramus width, ramus height and mechanobiological indicators (n=16, 8 males and 8 females). Solid lines represent curve fittings where the differences are statistically significant ($p < 0.05$), and dashed lines represent curve fittings where the differences are not statistically significant ($p \geqslant 0.05$). (**A**) Oxygen availability: correlations with 2D mandibular size, ramus width, and ramus height across bite forces of 11N, 30N, and 60N. (**B**) Glucose availability: correlations with 2D mandibular size, ramus width, and ramus height across bite forces of 11N, 30N, and 60N. (**C**) Lactate accumulation: correlations with 2D mandibular size, ramus width, and ramus height across bite forces of 11N, 30N, and 60N. (**D**) ATP production: correlations with 2D mandibular size, ramus width, and ramus height across bite forces of 11N, 30N, and 60N. Statistical details including p-values and R^2 are provided in **Supplementary Table 7**

Supplementary Figure 7: Mechanistic Insights: Mandibular Size and Nutrient Availability

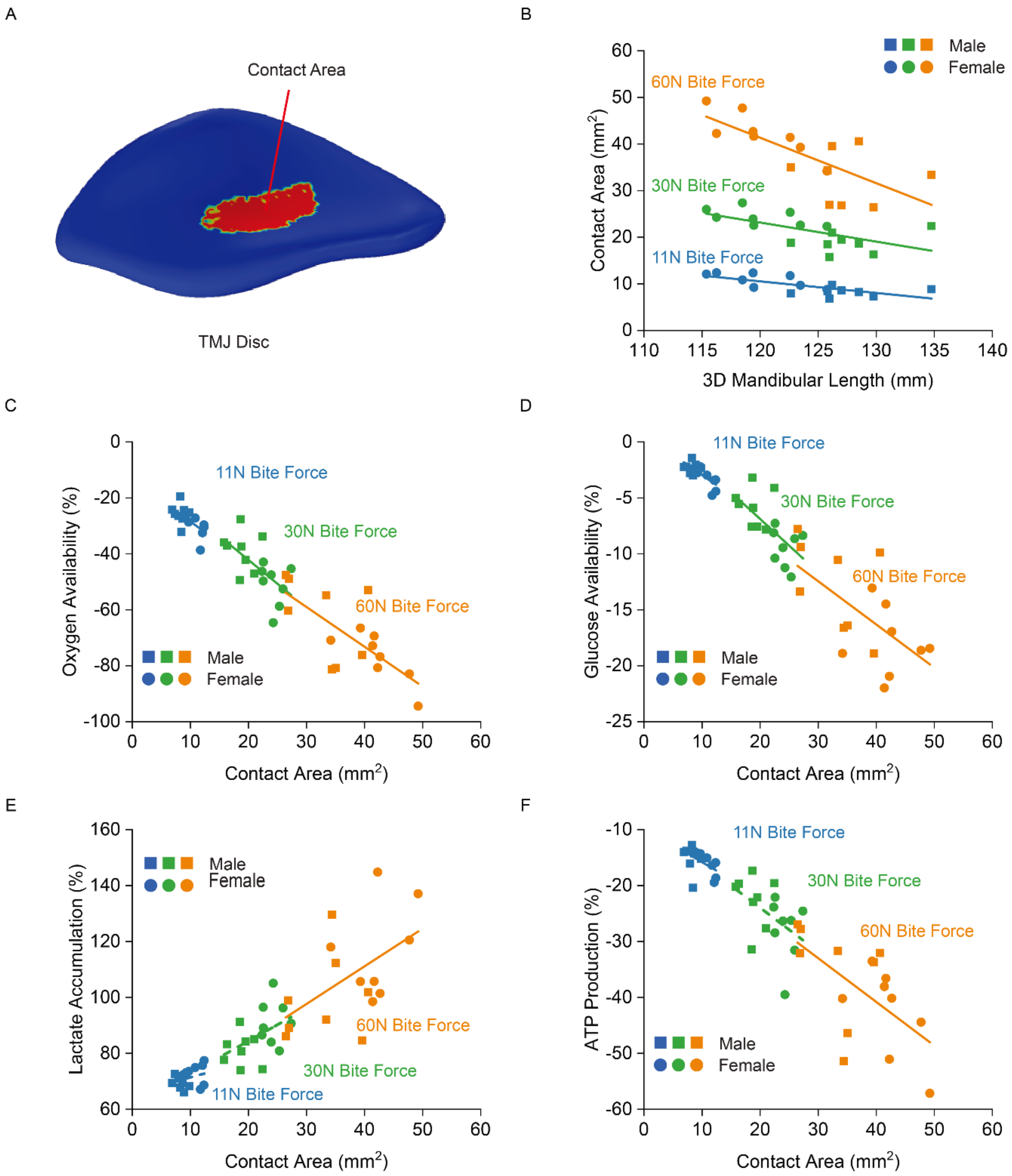

Mechanistic analysis for the relationship between mandibular size and nutrient availability (n=16, 8 males and 8 females). Solid lines represent curve fittings where the differences are statistically significant ($p < 0.05$), and dashed lines represent curve fittings where the differences

are not statistically significant ($p \geqslant 0.05$). (**A**) Contact area between the condyle and the disc. (**B**) 3D mandibular length is negatively correlated with contact area, indicating that subjects with large mandible size will have smaller condyle surface area with nutrient exchange blocked due to contact (11N: p=0.002, R^2=0.5134; 30N: p=0.010, R^2=0.3869; 60N: p=0.002, R^2=0.5250). (**C**) Relationship between contact area and oxygen availability (11N: p=0.009, R^2=0.3965; 30N: p=0.011, R^2=0.3829; 60N: p=0.001, R^2=0.5281). (**D**) Relationship between contact area and glucose availability (11N: p=0.001, R^2=0.5760; 30N: p=0.006, R^2=0.4315; 60N: p=0.010, R^2=0.3871). (**E**) Relationship between contact area and lactate accumulation (11N: p=0.258, R^2=0.0903; 30N: p=0.063, R^2=0.2255; 60N: p=0.038, R^2=0.2740). (**F**) Relationship between contact area and ATP availability (11N: p=0.047, R^2=0.2541; 30N: p=0.063, R^2=0.2263; 60N: p=0.012, R^2=0.3713).

Reference

1. Selvaraju RR, et al. *2017 IEEE International Conference on Computer Vision (ICCV)*. 2017:618-26.
2. Hill AV. The heat of shortening and the dynamic constants of muscle. *Proceedings of the Royal Society of London Series B-Biological Sciences.* 1938;126(843):136-95.
3. Venkatasubramanian R, et al. Incorporating energy metabolism into a growth model of multicellular tumor spheroids. *J Theor Biol.* 2006;242(2):440-53.
4. Wu Y, et al. Effect of Sustained Joint Loading on TMJ Disc Nutrient Environment. *J Dent Res.* 2019;98(8):888-95.
5. Kuo J, et al. Regional Cell Density Distribution and Oxygen Consumption Rates in Porcine TMJ Discs: An Explant Study. *Osteoarthritis and Cartilage.* 2011;19(7):911-8.
6. Urban JPG, et al. Nutrition of the Intervertebral Disk: An: In Vivo: Study of Solute Transport. *Clinical Orthopaedics and Related Research®.* 1977(129).
7. Lai WM, et al. A triphasic theory for the swelling and deformation behaviors of articular cartilage. *J Biomech Eng.* 1991;113(3):245-58.
8. Kuo J, et al. Effect of mechanical loading on electrical conductivity in porcine TMJ discs. *J Dent Res.* 2011;90(10):1216-20.
9. Wright GJ, et al. Effect of mechanical strain on solute diffusion in human TMJ discs: an electrical conductivity study. *Ann Biomed Eng.* 2013;41(11):2349-57.
10. Huang CY, and Gu WY. Effects of mechanical compression on metabolism and distribution of oxygen and lactate in intervertebral disc. *J Biomech.* 2008;41(6):1184-96.
11. Wu Y, et al. Effect of cartilage endplate on cell based disc regeneration: a finite element analysis. *Mol Cell Biomech.* 2013;10(2):159-82.

12. Nickel JC, et al. Tractional Forces, Work and Energy Densities in the Human TMJ. *Craniofac Growth Ser.* 2009;46:427-50.

13. Bower AF. *Applied mechanics of solids.* CRC press; 2009.

14. Ogden RW. *Non-linear elastic deformations.* Courier Corporation; 1997.

15. Sadd MH. *Elasticity: theory, applications, and numerics.* Academic Press; 2009.

16. Zhang F, et al. Mass properties of the human mandible. *Journal of Biomechanics.* 2002;35(7):975-8.

17. Koolstra JH, and van Eijden TM. Combined finite-element and rigid-body analysis of human jaw joint dynamics. *J Biomech.* 2005;38(12):2431-9.

18. Sélard E, et al. Finite element study of nutrient diffusion in the human intervertebral disc. *Spine (Phila Pa 1976).* 2003;28(17):1945-53; discussion 53.

19. Huang C-Y, and Gu WY. Effects of mechanical compression on metabolism and distribution of oxygen and lactate in intervertebral disc. *Journal of Biomechanics.* 2008;41(6):1184-96.

20. Yao H, and Gu WY. Physical Signals and Solute Transport in Cartilage Under Dynamic Unconfined Compression: Finite Element Analysis. *Annals of Biomedical Engineering.* 2004;32(3):380-90.

21. Zhu Q, et al. Cell viability in intervertebral disc under various nutritional and dynamic loading conditions: 3d finite element analysis. *J Biomech.* 2012;45(16):2769-77.

22. Hirose M, et al. Three-dimensional finite-element model of the human temporomandibular joint disc during prolonged clenching. *European Journal of Oral Sciences.* 2006;114(5):441-8.

23. Mori H, et al. Three-dimensional finite element analysis of cartilaginous tissues in human temporomandibular joint during prolonged clenching. *Archives of Oral Biology.* 2010;55(11):879-86.
